# Hyperspectral proximal sensing shows clear relation between Spatial pattern of leaf traits and bacterial alpha diversity

**DOI:** 10.1038/s41598-025-33183-4

**Published:** 2025-12-30

**Authors:** Fanhao Kong, Annabell Rosemarie Wagner, Susanne Walden, Eric Martiné, Sebastian Achilles, Lucy Saueressig, Stella Drechsler, Lars Opgenoorth, Robert R. Junker, Hamed Azarbad, Mona Schreiber, Maaike Y. Bader, Jörg Bendix

**Affiliations:** 1 Laboratory for Climatology and Remote Sensing, Department of Geography, Marburg University, 35032 Marburg, Germany; 2 Marburg University, Department of Biology, Evolutionary Ecology of Plants, 35043 Marburg, Germany; 3 Department of Biology, Plant Ecology and Geobotany, Marburg University, 35043 Marburg, Germany; 4 Department of Geography, Ecological Plant Geography, Marburg University, 34032 Marburg, Germany; 5 Department of Biology, Marburg University, 35043 Marburg, Germany

**Keywords:** Ecology, Ecology, Environmental sciences, Plant sciences

## Abstract

The phyllosphere bacteria play a crucial role in global greenhouse gas emissions and sequestration, but the spatial interactions between phyllosphere bacterial diversity, host leaf traits and environmental variation remain poorly understood. This gap is mainly due to methodological limitations in linking the spatial pattern of bacterial diversity to leaf traits. Here, we present machine learning models based on visible and near-infrared (VIS-NIR) leaf hyperspectral proximal sensing that are used to independently predict the phyllosphere bacterial alpha-diversity indices and leaf traits under different abiotic conditions for both sides of the leaf. We demonstrate that the models can effectively represent leaf traits and bacterial alpha-diversity indices for different abiotic environmental conditions. The cross-correlation of the spatial patterns as a result of the spatial application of the independent models reveals fine-scale associations between leaf traits and bacterial colonization patterns. Our findings highlight the great potential of hyperspectral proximal sensing for understanding the relationship between leaf bacterial richness and leaf resources within the leaf microecosystem. Ultimately, this will enhance our capacity to quantify the contribution of the leaf bacteria to the greenhouse gas balance of the forest canopy in a changing climate.

**Supplementary Information:**

The online version contains supplementary material available at 10.1038/s41598-025-33183-4.

Addressing climate change mitigation demands not only reducing atmospheric greenhouse gas (GHG) concentrations but also enhancing the capacity of ecosystems to regulate GHG fluxes.

Phyllosphere bacteria specifically participate in key ecological processes — contributing to carbon–nitrogen–phosphorus (C–N–P) cycling^1^, modulating foliar methanol emissions, fixing atmospheric nitrogen, and promoting phosphate solubilization^2–5^ — all relevant to the production and consumption of key GHGs such as carbon dioxide (CO_2_), methane (CH_4_), and nitrous oxide (N_2_O)^6^. The phyllosphere is an open and globally extensive habitat, and the total terrestrial phyllosphere area is estimated to be around 6.4 × 108 km^27^. It provides multiple vital microhabitats for bacteria at the terrestrial atmosphere–ecosystem boundary. Owing to the vast bacterial populations hosted by temperate foliage, the worldwide phyllosphere bacterial population is estimated to be as high as 10^26^ cells^7^. At this scale, even small-scale processes at the leaf–atmosphere interface can influence atmospheric composition. However, we currently lack tools for spatially explicit microbial ecology across leaf-to-landscape scale^8^, limiting our ability to inform microbial parameters relevant to canopy photosynthesis and respiration.

Furthermore, the global scale of phyllosphere leaf area is approximately twice that of the land surface area^9^. The leaf serves as a unique micro-ecosystem, supporting a diverse community of bacteria that exhibit remarkable biochemical and physiological diversity^9^. In addition to exploring the ecological importance of microbial activities related to GHGs, it is also crucial to systematically investigate the bacterial diversity spatial variation across various parts of the leaf. Notably, while bacteria depend on leaf resources for survival, they can also actively alter leaf traits, influencing nutrient dynamics, structure, and physiology^10,11^. For example, phyllosphere nitrogen availability has been shown to shape bacterial community structure, while cyanobacteria have been reported to contribute significantly to host leaf nitrogen budgets by fixing atmospheric nitrogen^12^. Therefore, it is also of interest to investigate the distribution of these leaf traits across the leaf surface, as they provide key insights into the structure and functional potential of the phyllosphere bacteria.

A better understanding of the interactions between bacteria-relevant leaf traits and bacterial communities can inform how these interactions influence plant health, nutrient cycles, and ecosystem functioning, especially when we upscale everything from leaf to canopy level and ultimately to the forest ecosystem level. This understanding can improve management decisions aimed at enhancing ecosystem stability and mitigating the negative effects of climate change^13^.

Although there is an urgent need to discover these intercorrelations, the lack of advanced data collection technologies has, for a long time, limited our analysis of leaf traits and phyllosphere bacteria^8^. Recent advances in hyperspectral imaging and proximal sensing technologies have enabled for tracking different plant traits across a wide spectral range^14–16^. These methods have been applied to detect physiological shifts, disease symptoms, and biochemical changes in plant tissues by scanning bulk samples or whole plants^17,18^. However, their application to phyllosphere bacteria remains largely unexplored. Bacterial communities present particular challenges for remote sensing due to their microscale heterogeneity, environmental sensitivity, and the absence of non-invasive, high-resolution technologies to capture bacterial signals from host leaf surfaces.

In this study, we integrate hyperspectral proximal sensing with machine learning to investigate spatial patterns of phyllosphere bacterial α-diversity and their interactions with leaf traits. We selected Quercus robur (English oak) as the model species because it is a widespread, dominant.

deciduous tree with pronounced within-leaf and seasonal heterogeneity. We conducted seasonal field campaigns in the Marburg Open Forest (Supplementary Figs. 1–2; Supplementary Text 1) to collect data on leaf spectral profiles, physiological traits, and bacterial communities from both leaf surfaces under varying light conditions. Our central hypothesis is that leaf traits and the phyllosphere bacterial community can be captured via hyperspectral reflectance and machine learning, and that the two are tightly associated. Here we focus on the phyllosphere bacterial community, profiled by 16 S rRNA amplicon sequencing; eukaryotic members of the leaf microbiome (e.g., fungi, yeasts, algae, protists) were not assessed in this study. We: (1) independently trained predictive machine learning models to estimate bacterial α-diversity indices and leaf traits from hyperspectral data; (2) mapped spatial distributions of bacteria and trait variables across leaf surfaces; and (3) examined leaf-level trait–bacteria correlations to reveal the ecological interactions shaping phyllosphere community structure. Our approach offers new insights into the spatial bacterial ecology of the phyllosphere and provides a foundation for quantifying bacterial contributions to canopy-level GHG exchange in forest ecosystems under changing environmental conditions.

## Methods

To capture microhabitat-driven variation, we collected both sunlit and shaded leaves from the same individual trees, reflecting distinct environmental gradients^19^. For each leaf, we scanned both adaxial and abaxial surfaces to account for their anatomical and functional differences. For each leaf surface we averaged reflectance across all valid pixels at each of the 119 spectral bands, yielding one band-wise mean per surface (i.e., two mean spectra per leaf). These surface-level spectra were then used as predictors in machine-learning models targeting leaf traits and bacterial α-diversity indices (each surface treated as an observation linked to the corresponding leaf- level response). For mapping, we applied the trained model to every pixel spectrum on selected leaves/surfaces to obtain pixel-wise predictions. We then conducted leaf-level Spearman correlation analyses to investigate trait–bacteria relationships at fine spatial resolution.

### Sampling design

Fieldwork required handling leaf samples as sterile as possible. In the forest, all the leaves were collected by professional climbers. All researchers in the forest wore surgical masks and gloves, and only handled the leaves with ethanol disinfected tweezers. We conducted field campaigns in July and September 2023, each lasting for four days (17–20 July and 25–28 September). Measurements were made between 10 AM and 2 PM local time under dry conditions without rainfall. We sampled the same 8 Quercus robur trees for each field campaign, selecting three sunlit branches and three shade branches per tree. Sunlit branches were chosen from the top canopy, on the southern side and in the periphery, while shade branches were selected from the lower canopy, on the northern side and near the stem. From each branch, we collected four leaves, yielding a total of 12 sunlit leaves and 12 shade leaves per tree. After collection, each sample was immediately placed in a Petri dish, secured with a rubber band, sealed inside a plastic bag, and then stored in cooling boxes to preserve conditions. In total, 384 leaf samples were collected across different seasons and light conditions. For leaf traits measurement, each season included 96 sunlit and 96 shade leaves, with a quarter of each (24 leaves) further analyzed for bacterial diversity.

### Hyperspectral imaging and preprocessing

Leaf reflectance spectra were acquired using OCI-1000 Series Hyperspectral Imagers (BaySpec, Incorporated, San Jose, California 95131 USA), which provide continuous spectral coverage across 119 wavelengths in the visible to near-infrared (VIS-NIR) range (470–980 nm) with spectral resolution < 5 nm FWHM)^20^. The OCI-1000 uses true push-broom scanning and features automated data capture with variable scan speeds. Imaging was conducted using a calibrated artificial light source and a grey reference spectralon board (50% reflectance). Before each daily field campaign, scans of the white standard and a dark background (0% reflectance, achieved by covering the lens) were performed to calibrate reflectance measurements. Hyperspectral imaging was conducted immediately after leaf collection in the field. Processing occurred in the lab, where we excluded shade leaf samples from Tree 5 (September sampling) due to high surface moisture from fog, which introduced spectral artifacts.

Raw data processing followed three main steps. First, for each leaf, we independently generated hypercube files for the adaxial and abaxial surfaces using BaySpec’s Cube Creator, selecting the required spectral stripes to ensure full coverage of the leaf area^21^. Second, leaf regions were extracted from the hypercubes. Since raw scans include background pixels, we applied an NDVI- based mask (computed from red and NIR bands^22^ ) to isolate green vegetation. Where NDVI alone was insufficient — e.g., in yellowed or veined areas — additional reflectance-based thresholds were applied to refine segmentation. Each image contained up to 2048 × 1600 spatial pixels, with imaging conducted at 20 cm distance, corresponding to 30.93 μm spatial resolution of a pixel. After background removal, each leaf surface retained hundreds of thousands of valid pixels. For each leaf surface (adaxial and abaxial), pixel spectra were then processed separately and averaged to yield one surface-level reflectance spectrum per side. The final step involves further smoothing the data to identify potential outliers and enhance spectra data quality. First-order derivatives were computed to amplify spectral variation and identify anomalous samples. A Savitzky-Golay (SG) filter^23^ was then applied to reduce noise while preserving key spectral features. The derivative of a spectrum represents its rate of change with respect to wavelength, enabling components to be more distinct compared to the original (zero-order) spectrum^24^ (Supplementary Fig. 3). Outlier detection removed three samples from July and four from September. For smoothing, the SG filter used a window size of 4 (July) or 3 (September) with a polynomial order of 1 in both cases.

### Leaf traits data

Leaf traits and corresponding measurement units are detailed in Supplementary Table 1. Chlorophyll content (Chl), flavonoid content (Flav), anthocyanin content (Anth), and Nitrogen Balance Index (NBI) were estimated non-destructively using a Dualex device. NBI is an indicator of the nitrogen status of the leaf, showing a direct correlation with the bulk nitrogen content^25,26^. Leaf area was calculated from the images captured by a digital camera (Canon EOS 60D), with analysis performed in ImageJ software^27^. Fresh mass (FM) of the collected leaf samples was measured immediately after collection. After drying the samples at 50℃ for 48 h, the dry mass (DM) was measured. Leaf Mass per Area (LMA) was then calculated as the ratio of DM to leaf area, using the formula:

1 $$LMA=\frac{{DM}}{{Leaf{\kern 1pt} Area}}$$

Leaf Water Content (LWC) was derived as a percentage of FM using the formula^28^:

2 $$LWC=100 \times \frac{{FM - DM}}{{FM}}$$

To determine leaf carbon (C) and nitrogen (N) content, samples were processed as follows: Leaves were dried overnight in Heraeus Drying cabinet UT 6060. Subsequently, for each leaf, 25 mg of material from the tip was individually collected and placed in a separate centrifuge tube. Each leaf subsample was then shredded for 3 min using Retsch Mixer Mill MM 301. From the shredded material, a 2–5 mg portion from each subsample was individually taken and transferred into a separate small aluminum container for further analysis. We used Vario El Cube elemental analyzer (Elementar GmbH) to measure the C and N content of the samples. C and N content data are only available for leaf samples collected in September.

### Bacterial α-diversity indices

Epiphytic bacterial communities were collected by washing leaf samples in 20 ml phosphate buffer saline (RotiFair PBS 7.4, Carl Roth, Karlsruhe, Germany) while applying high-frequency vibration (Pulsifier II PUL200, Microgen Bioproducts Ltd, Surrey, UK). The suspension was centrifuged (7000xg, 20 min, 4 °C), and the pellet was resuspended in 2 ml supernatant by vortexing. One ml of the concentrated suspension was shock-frozen in liquid nitrogen and stored at -80℃ until DNA extraction. For 16 S Amplicon sequencing 1 sample per branch per each sampling season was randomly selected. We extracted DNA using the DNeasy Plant Pro Kit (Qiagen, Hilden, Germany) to minimize host plant DNA contamination. One ml of suspension was transferred into a tissue disruption tube with 500 µl lysis solution and homogenized using a mill (30/s, 10 min). LGC Genomics (Berlin, Germany) amplified (primers: 799 F (AAC MGG ATT AGA TAC CCK G), 1115R (AGG GTT GCG CTC GTT G)) and Illumina MiSeq sequenced the V5–V6 region of the 16 S rRNA gene to identify bacterial ASVs. Raw sequencing data were processed using the open-source platform ”QIITA”^29^ and QIIME2^30^. In quality filtering, samples below the median species richness saturation point were excluded to ensure excluding of low-quality samples. This process was performed using the rtk package (version 0.2.6.1)^31^. Subsequently, contaminant ASVs from the orders Chloroplast, Rickettsiales, and the phyla Cyanobacteria and Chloroflexi.

were removed. Additionally, ASVs appearing in fewer than two samples were excluded. We rarefied the data to the minimum sequencing depth observed among samples using the package rtk (version 0.2.6.1) with 99,999 iterations to correct for differences in sequencing depth. Alpha diversity metrics—including richness, Shannon diversity index, inverse Simpson index, and Chao1 estimates — were calculated as the mean values across all rarefaction iterations for each sample. To estimate the number of rare microbial taxa, we used the difference between Chao1 and richness as a proxy. For quality filtering and alpha diversity calculation, we used R version 4.3.2 (R Core Team, 2023). Supplementary Table 1 lists the microbiome indices we measured for each leaf sample in this study, together with their units.

### Machine learning models for traits and bacteria

To relate leaf traits and bacterial α-diversity with measured leaf spectra, we developed two kinds of ML models: the individual PLSR model and the stacking regression model. The leaf-level hyperspectral reflectance (470–980 nm, VIS-NIR) detected by our hyperspectral camera served as predictor variables. The target variables included leaf traits and bacterial α-diversity indices (Supplementary Table 1) derived per leaf. Leaf traits and bacterial α-diversity indices were modeled using two fully independent sets of machine learning models, each trained and validated separately. Since seasonal variations can influence phyllosphere conditions, our ML models were applied separately to the two growing seasons for leaf traits variables. However, due to the limited sample size for bacterial variables, a single combined dataset comprising all seasons was used to develop the ML models. Prior to training the chemical nitrogen (N), carbon (C), and the C/N ratio models, both the hyperspectral predictors and traits were log-transformed to normalize their distributions. In addition, separate ML models were constructed for the spectra of sunlit leaves, shade leaves, and the adaxial and abaxial surfaces of the leaves.

The hyperspectral data included 119 narrow spectral bands with high collinearity. To address this, Partial Least Squares Regression (PLSR) model was employed to reduce dimensionality and mitigate multicollinearity by creating a smaller number of latent variables that carry the most important information while avoiding overfitting^32^. This approach has been widely used to explore the relationship between leaf physiochemical traits and spectral reflectance^33,34^.

However, PLSR alone may not always fully capture complex nonlinear relationships between leaf traits, microbiome indices, and spectral data, which may limit its predictive accuracy in certain cases. To address this limitation, alternative machine learning methods, including Support Vector Regression (SVR) and Random Forest (RF), could be employed to better capture nonlinear relationships. Therefore, in order to take advantage of the strengths of multiple regression approaches, an ensemble learning strategy was applied. Stacking regression has proven to be a suitable approach for integrating different models, allowing for improved predictive performance by combining the outputs of individual base models. Stacking regression consists of two levels: in the first level (level-0), multiple base-models are independently trained. In the second level (level-1), a single meta-model is trained using the predictions from the base-models as input, producing the final output with improved prediction accuracy^35^. For this study, we ensembled PLSR and SVR as level-0 base-models, and RF as level-1 meta-model to generate the final predictions.

Moreover, due to the high-dimensional spectral data and a limited number of leaf samples, we first applied the PLSR model for dimensionality reduction. Then we used the selected smaller set of latent components as predictors also in another base-model (SVR model) to prevent overfitting. In this study, machine learning models were subjected to five-fold cross-validation to avoid overfitting and grid search to optimize hyperparameters. For the models, variable importance was assessed using Variable Importance in Projection (VIP) scores, which quantify the contribution of each spectral feature to the predictive performance. Here, accuracy metrics were used to evaluate the degree of fitness between the test dataset of the predictor variables and the predicted dataset of the response variable. The expressions for each metric are as follows:

1. Root-mean-square error (RMSE) .

The Root Mean Squared Error (RMSE) measures the average difference between model- predicted values and the actual values^36^. It can estimate how accurate the model is able to predict the target values. The lower the RMSE, the better the model is.

2. Coefficient of determination (R^2^).

The proportion of the variance in the response variable that is predictable from the predictor variables^37^. R^2^ has an upper bound of 1, which represents a perfect fit. On the other hand, R^2^ has no lower bound, where a value of 0 representing the trivial fit provided by the horizontal line y = K, where K is the mean of the target values across all training data points. Any negative value of R^2^ indicates a poorer fit compared to the horizontal line y = K^38^. The formula of the R-squared is defined as:

3 $${R^2}=1 - \frac{{{\sum _i}{{(yi - fi)}^2}}}{{{\sum _i}{{(yi - \bar {y})}^2}}}$$

in which yi represents the observed value for the i^th^ observation, $\bar{y}$ is the mean of all actual values in the testing dataset and f_i_ is the predicted value for the i^th^ observation.

### Spatial distribution maps and area-wide cross-correlation

We applied the optimal ML models to predict the specific leaf traits and bacterial α-diversity indices for each pixel within the selected leaf region. In hyperspectral images, pixels with similar spectral patterns will yield comparable predicted values, which are subsequently visualized using comparable colors in the resulting spatial distribution maps^39^. Since the reflectance spectrum of individual pixels typically contains small-scale fluctuations and lacks smoothness, we first applied a Savitzky–Golay (SG) filter to smooth each pixel’s spectral curve. Then, a 5 × 5 window moving average filter^40^ was appied to further remove spatial noises.

To investigate the relationship between leaf traits and bacterial α-diversity indices at the leaf level, we conducted a Spearman correlation analysis within the selected leaf region. First, we applied trained PLSR models for various variables, including Chl, NBI, Anth, N, C, C/N, richness and Chao1, to all pixels within the selected leaf region. Then, we performed a Spearman correlation analysis based on the entire predicted leaf region to estimate the relationships between leaf traits and bacterial α-diversity indices within the phyllosphere, using Spearman correlation coefficients (ρ) as the measure of association. We specifically chose PLSR instead of the stacking model because, based on our spatial monitoring maps, PLSR is a more effective approach for capturing detailed and subtle variations.

In addition to the pixel-wise analysis, we also performed a leaf-based correlation analysis using direct measurements of bacterial α-diversity indices and leaf chemical traits. This analysis was conducted on 45 leaves for which both bacterial α-diversity indices (richness and Chao1) and chemical measurements (N and C contents) were available, collected during the September sampling campaign. For these leaves, Spearman correlation coefficients were calculated at the whole-leaf level to assess the broader trait–diversity relationships. This complementary correlation analysis allowed us to validate the pixel-level predictions and examine trait–bacterial α-diversity correlations across individual leaves based on ground-truth measurements. Some scientists^41,42^ have provided guidelines for interpreting correlation coefficients (Supplementary Table 2).

## Results

### Leaf trait models

**Fig. 1:**
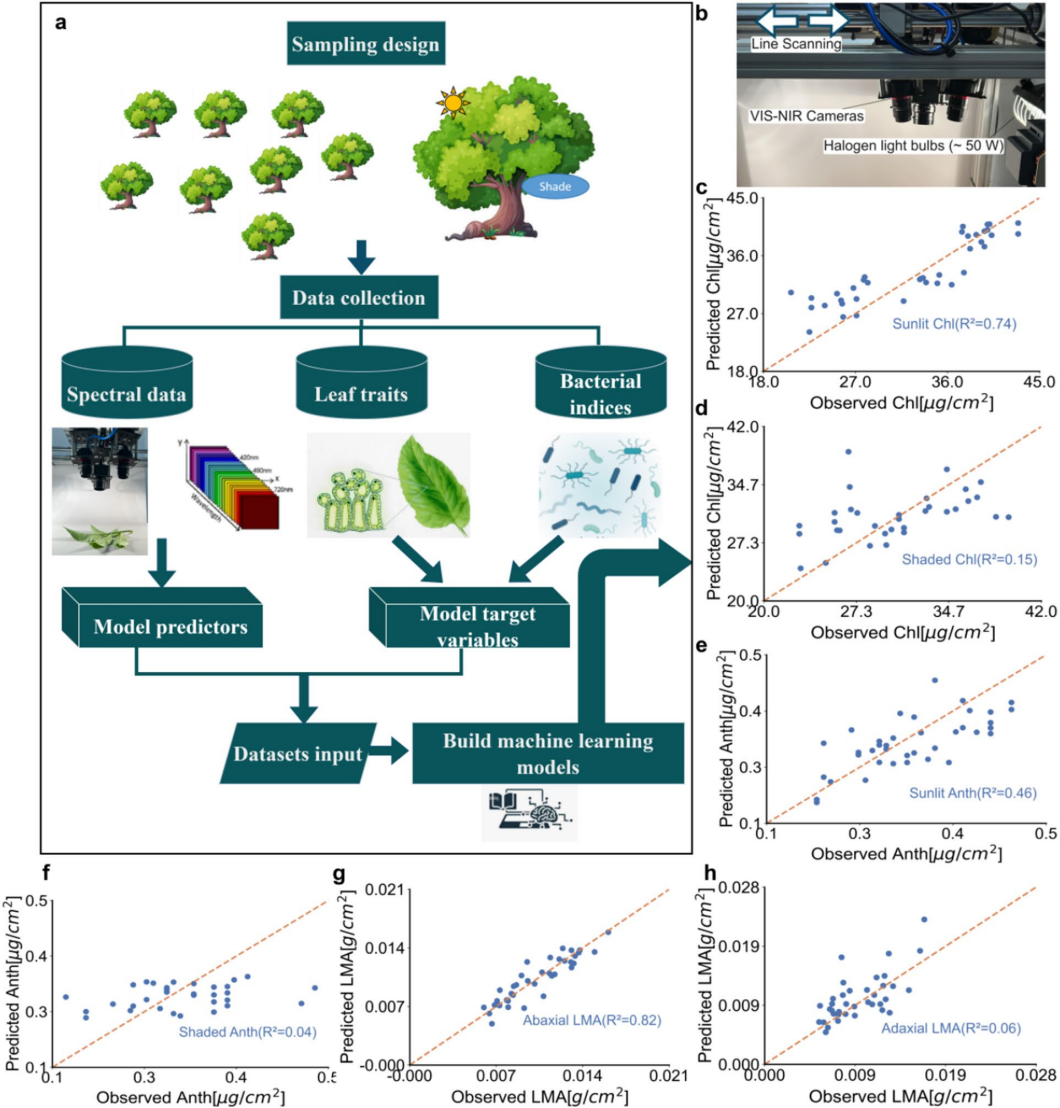
Machine learning framework for predicting phyllosphere bacterial diversity and leaf traits from hyperspectral data. **a**, Overview of the modeling pipeline. Leaf spectral data were used as predictors, while leaf physiochemical traits and bacteria diversity indices served as response variables. The workflow includes sampling design, collection of hyperspectral, traits, and bacteria data, and model development. **b**, Custom-designed OCI-1000 hyperspectral imaging system (470–980 nm) with calibrated illumination sources, optimized for proximal sensing of intact oak leaves under field conditions. **c-f**, Observed vs. predicted trait values based on PLSR models trained on leaf spectral data collected in September: **(c)** Sunlit chlorophyll content [µg/cm2 ] (Chl; R² = 0.74); **(d)** Shaded Chl (R² = 0.15); **(e)** Sunlit anthocyanin content [µg/cm2 ] (Anth; R² = 0.46); **(f)** Shaded Anth (R² = 0.04). **g**,** h**, Observed vs. predicted leaf mass per area (LMA) values based on PLSR models trained on leaf spectral data collected in July: **(g)** Abaxial leaf mass per area (LMA; R² = 0.82); **(h)** Adaxial LMA (R² = 0.06). Dashed orange line indicates 1:1 relationship for visual reference.

We acquired hyperspectral images of both the adaxial and abaxial surfaces of 365 Quercus robur leaves under varying environmental conditions. Pixel spectra were processed separately per leaf surface and averaged to yield one surface-level spectrum per side; these surface-level spectra were then used as predictors to independently predict leaf-level physicochemical traits and bacterial diversity indices (Fig. 1a, Supplementary Table 1).

Partial least squares regression (PLSR) and stacking ensemble models produced comparable accuracy for most leaf trait predictions (Table 1). Across seasons, models trained on chemical traits performed more reliably in September than in July, particularly when sunlit spectra were used as input features. Notably, chlorophyll (Chl; R^2^ ≥ 0.74) and anthocyanin (Anth; R^2^ ≥ 0.46) predictions reached higher accuracy under sunlit conditions in September (Fig. 1b, c). Furthermore, abaxial spectra generally showed a stronger connection than adaxial spectra to structural (morphological) traits such as LMA and LWC in both seasons. The strongest prediction was observed for LMA in July using abaxial spectra (PLSR R^2^ = 0.82). However, several key variations were observed. Stacking models generally underperformed relative to PLSR when using shade spectra, often resulting in lower R^2^ values. Moreover, stacking models also showed unstable performance when predicting structural (morphological) traits.

**Fig. 2:**
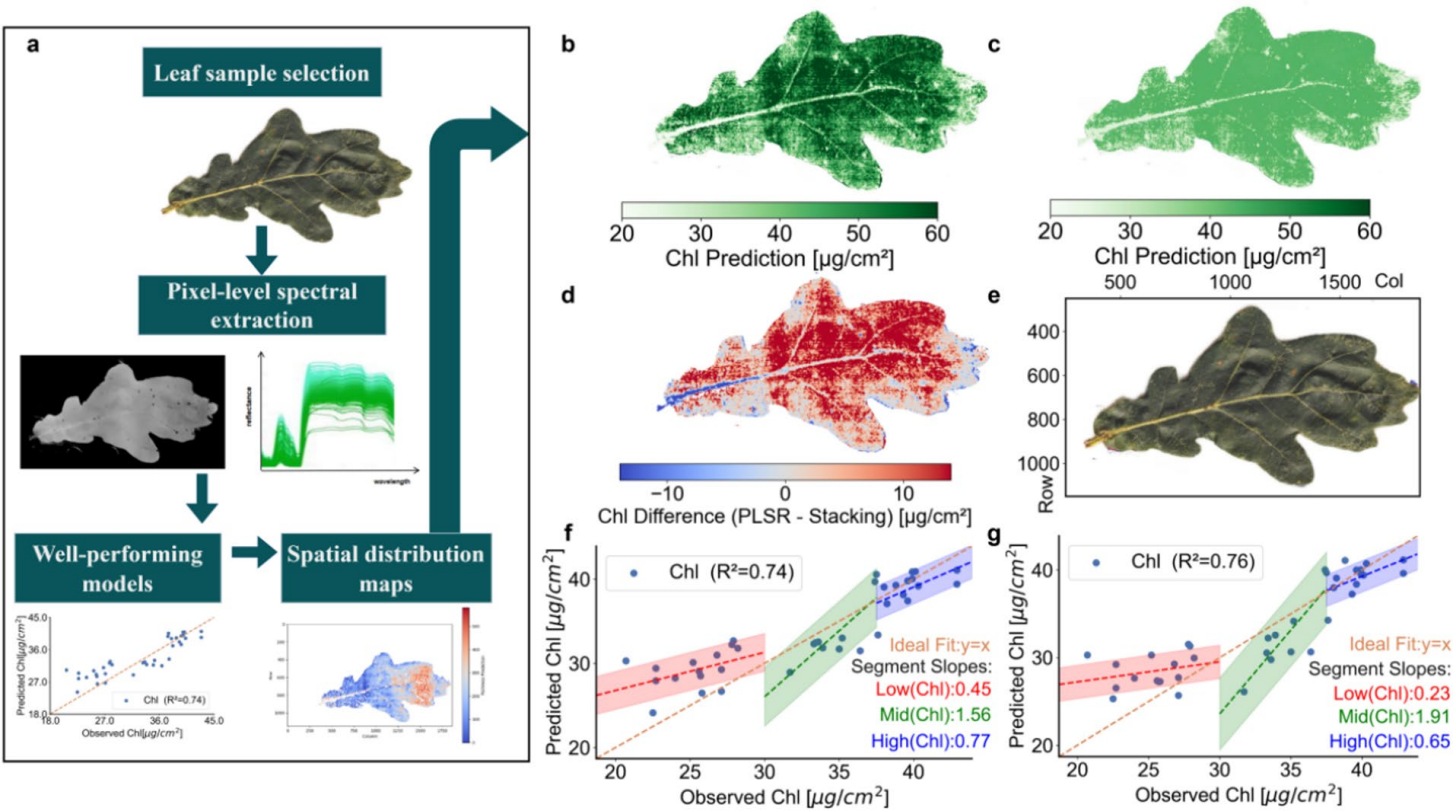
Spatial prediction of chlorophyll content and model comparison using hyperspectral imaging and machine learning. **a**, Schematic of the workflow for applying machine learning models to predict spatial distributions of leaf traits and microbiome diversity indices, and for conducting trait–microbiome cross-correlation analysis. **b-d**, Predicted chlorophyll (Chl) content [µg/cm^2^ ] across a sunlit Quercus robur leaf collected in late August 2024, based on models trained on September sunlit spectra (PLSR: R^2^ = 0.74, Stacking: R^2^ = 0.76) **(b)** Spatial predictions from the PLSR model **(c)** Spatial predictions from the stacking model **(d)** Difference map between PLSR and stacking predictions (PLSR minus stacking). Red regions indicate higher Chl values predicted by the PLSR model, blue regions indicate higher predictions by the stacking model, and white regions indicate minimal difference. **e**, RGB image of the same leaf, providing a visual reference for structural and pigmentation features. **f**,** g**, Observed vs. predicted Chl content for **(f)** the PLSR model and **(g)** the stacking model. Data points are grouped by chlorophyll concentration: low (< 30 µg/cm2 ; red dashed fit), medium ( 30–37.5 µg/cm2; green dashed fit), and high (> 37.5 µg/cm2; blue dashed fit). Orange dashed line indicates the ideal 1:1 relationship.

We used Chl prediction as an illustrative example to demonstrate the spatial mapping potential of our models, applying the best-performing models to a high-resolution scan of a mature oak leaf collected under sunlit conditions in late August 2024. Using both the PLSR (R^2^ = 0.74) and stacking (R^2^ = 0.76) models trained on September data, we generated spatial maps of Chl content across 626,533 pixels (Fig. 2b, c). To compare pixel-wise model performance, we generated a difference image by subtracting stacking model predictions from PLSR predictions (Fig. 2d). An RGB image of the same leaf (Fig. 2e) was used as a qualitative reference, based on the known relationship between darker green coloration and elevated chlorophyll levels^43^. Finally, we evaluated the predictive performance of each model across three chlorophyll concentration intervals: low (< 30 µg/cm2), medium ( 30–37.5 µg/cm2), and high (> 37.5 µg/cm2 ). Observed vs. Predicted relationships for each model are shown in Fig. 2f, g, with linear fits indicating model robustness across the chlorophyll gradient.

### Leaf bacterial α-diversity models

Among the diversity indices, species richness and Chao1 showed better performance, especially when using shade-derived spectra as predictors (Table 2). While both richness and Chao1 quantify α-diversity based on observed taxonomic units, Chao1 further estimates the number of undetected rare taxa by incorporating singleton and doubleton counts. PLSR models achieved performance of R^2^ = 0.54 (richness) and R^2^ = 0.71 (Chao1),with stacking models yielding comparable results (richness: R^2^ = 0.51, Chao1: R^2^ = 0.56). In ecology, R^2^ values in the 0.20–0.50 range are commonly regarded as acceptable to good for complex systems, because these systems are very complex with many confounding factors blurring variance explanation of models^44,45^. By contrast, models trained to predict Shannon diversity, Evenness, and inverse Simpson indices performed poorly across all spectral inputs, reflecting lower spectral sensitivity to these community-level metrics (Table 2).

**Fig. 3:**
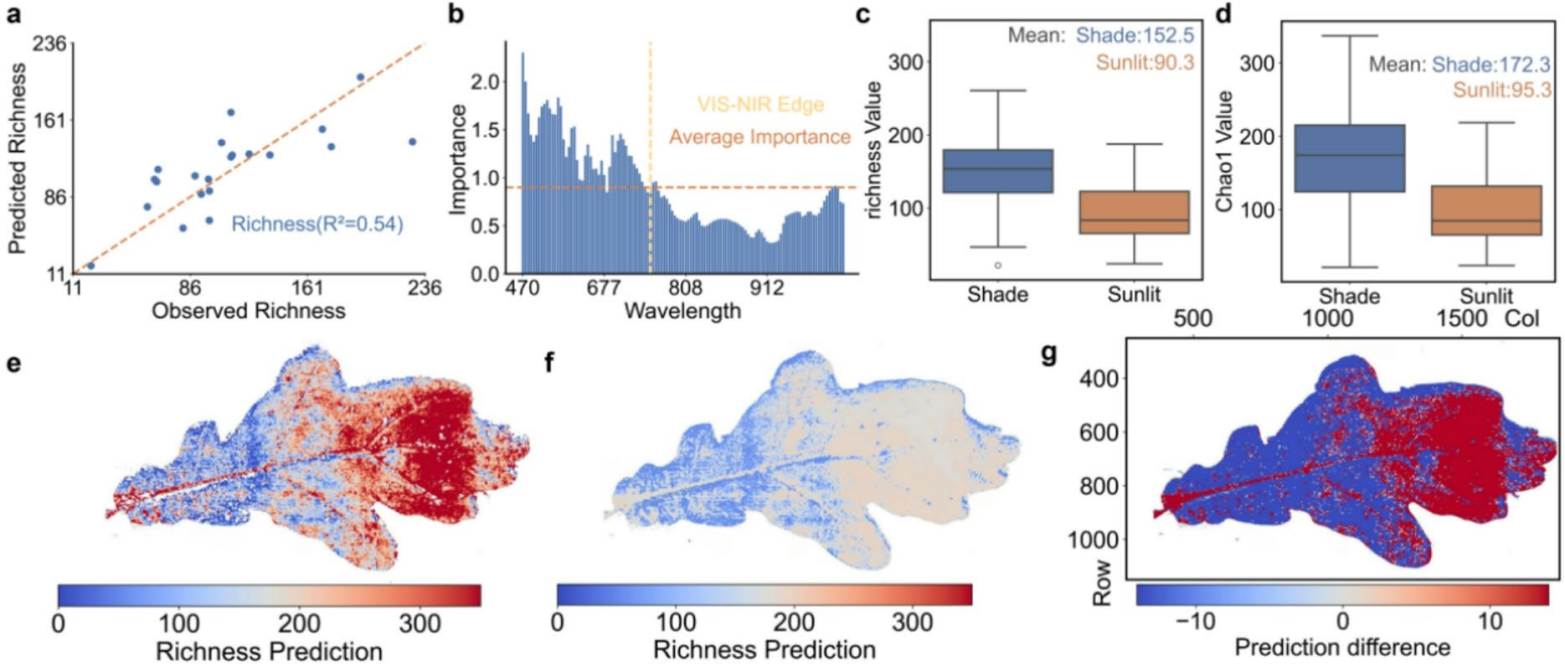
Prediction and spatial mapping of phyllosphere bacterial richness using hyperspectral models. **a**,** b**, Performance of the PLSR model for predicting bacterial species richness from shade leaf spectra (R^2^ = 0.54), as shown in Table 2**(a)** Scatterplot of observed versus predicted richness values. The dashed orange line indicates the ideal 1:1 relationship **(b)** Feature importance plot showing the relative contribution of individual spectral bands to the model. Blue bars represent the importance of each wavelength, with the orange dashed line indicating the average contribution. The vertical yellow dashed line demarcates the transition from the visible (VIS) to near- infrared (NIR) spectral regions. **c**,** d**, Comparison of observed bacterial α-diversity indices between sunlit (orange) and shaded (blue) leaves **(c)** Boxplot of species richness, defined as the total number of distinct microbial taxa detected per leaf **(d)** Boxplot of Chao1, a richness estimator that accounts for rare taxa. **e-g**, Spatial prediction of relative bacterial richness across a sunlit oak leaf sampled in late August 2024, using models trained on shade spectra (PLSR: R^2^ = 0.54, Stacking: R^2^ = 0.51) **(e)** Richness map based on the PLSR model **(f)** Richness map based on the stacking model **(g)** Difference image (PLSR minus stacking). Red areas indicate higher predictions from the PLSR model; blue areas indicate higher stacking predictions; white areas represent close agreement between models.

Scatterplots and feature importance values for the PLSR model predicting bacterial species richness from shade spectra are shown in Fig. 3a, b. The feature importance values fluctuate.

across the spectrum, with some peaks in both the Visible (VIS, 279 400–700 nm) and Near-Infrared (NIR, 950–980 nm) regions. This spectral importance pattern differs substantially from the trait models (Supplementary Fig. 3), where Anth and N showed sharp peaks in the visible region and around the VIS–NIR edge (approximately 700–750 nm).

Given the differences in model performance between sunlit and shaded leaves, we compared the observed values of species richness and Chao1 across these light environments (Fig. 3c, d). In both cases, shaded leaves showed substantially higher values than sunlit leaves (mean richness: 152.5 vs. 90.3; mean Chao1: 172.3 vs. 95.3).

Performance was also compared across abaxial and adaxial spectral inputs. In both richness and Chao1 predictions, abaxial spectra consistently yielded higher R^2^ values than adaxial spectra. For example, in predicting species richness, PLSR and stacking models achieved R^2^ values of 0.17 and 0.31, respectively, on abaxial spectra, compared to 0.07 and − 0.08 on adaxial spectra. This trend was consistent across both diversity metrics and model types.

As an example, we applied the richness prediction model (trained on shade spectra) to the same oak leaf used for chlorophyll mapping, enabling spatial visualization of bacterial richness across the leaf surface (Fig. 3e–g).

### Correlations of spatial traits and bacterial α-diversity patterns

**Fig. 4:**
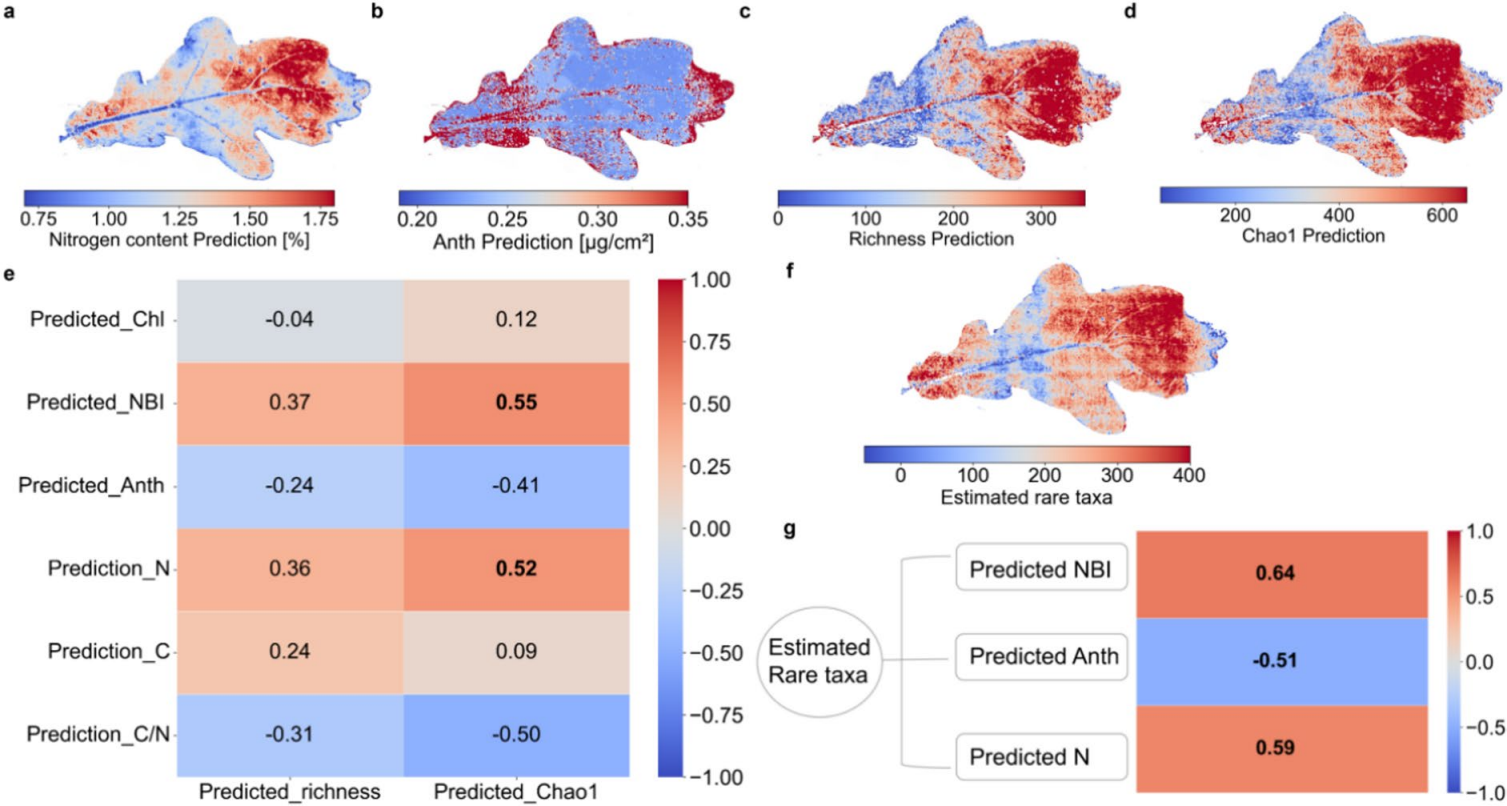
Spatial mapping and cross-correlation of phyllosphere bacterial α-diversity and leaf traits. **a**,** b**, Spatial predictions of leaf chemical traits from the PLSR model trained on sunlit spectra collected in September, applied to a sunlit Quercus robur leaf sampled in late August 2024. **(a)** Predicted Nitrogen content **(b)** Predicted anthocyanin (Anth) concentration. **c**,** d**, Spatial predictions of bacterial α-diversity indices from PLSR models trained on shade spectra. **(c)** Predicted bacterial species richness across the leaf surface. **(d)** Predicted bacterial Chao1 index, representing estimated richness including rare taxa. **e**, Spearman correlation matrix showing pairwise relationships among predicted leaf traits and bacterial α-diversity indices across the same individual leaf. Trait models included chlorophyll (Chl), nitrogen balance index (NBI), anthocyanin content (Anth), nitrogen (N), carbon (C), and carbon-to-nitrogen ratio (C/N). Bacterial models included species richness and Chao1. Positive correlations are shown in red, negative in blue; color intensity reflects correlation strength (scale: -1 to 1). Correlations with an absolute value greater than 0.5 are bolded, indicating strong associations. **f**, Spatial map of rare taxa count across the leaf surface, derived as the difference between predicted Chao1 and richness indices (Chao1 - Richness). **g**, Spearman’s correlation between rare taxa count and predicted leaf traits, including nitrogen balance index (NBI), anthocyanin content (Anth), and nitrogen content (N). Positive correlations were observed for NBI and N, and a negative correlation for Anth.

Figure 4a-d show the spatial distribution of PLSR predictions for Nitrogen content (R^2^ = 0.49) and Anth (R^2^ = 0.46) from sunlit spectra in September, while species richness (R^2^ = 0.54) and Chao1 (R^2^ = 0.71) were predicted using the PLSR model based on shade spectra for the selected leaf. Spatial prediction maps revealed distinct trait–bacterial α-diversity relationships across the leaf surface. Notably, predicted nitrogen (N) content (Fig. 4a) showed a clear spatial gradient, with higher values concentrated in the upper portion of the leaf. In contrast, anthocyanin (Anth) content exhibited an opposing pattern (Fig. 4b), with elevated Anth levels observed in regions of low N, particularly toward the leaf base and margins. The predicted bacterial richness and Chao1 indices (Fig. 4c, d) closely followed the spatial distribution of N content. This interpretation was further supported by spatial cross-correlation analysis (Fig. 4e), which showed positive Spearman correlation coefficients (ρ) between predicted N and both richness (ρ = 0.36) and Chao1 (ρ = 0.52). In contrast, predicted Anth was negatively correlated with bacterial α-diversity indices, particularly Chao1 (ρ = -0.41). Notably, the correlation between predicted N and Chao1 exceeded the threshold for a strong association (|ρ| > 0.5; Supplementary Table 2).

Since Chao1 showed stronger correlations with predicted N and Anth compared to richness, and given that Chao1 differs from richness primarily by accounting for rare taxa, which often represent highly specialized or environmentally sensitive members of bacterial communities^46^, we next examined the spatial distribution of these rare bacterial taxa in greater detail. To this end, we computed the difference between predicted Chao1 and richness values as a proxy for rare taxa count. The resulting spatial map (Fig. 4f) was closely aligned with the spatial patterns of predicted richness and Chao1 diversity indices, and further revealed that rare taxa followed a similar distribution to N content, with higher values in N-rich regions. Cross-correlation analysis confirmed this pattern, showing strong positive correlations between rare taxa and predicted N (ρ = 0.59) and NBI (ρ = 0.64), and a negative correlation with predicted Anth (ρ = -0.51) (Fig. 4g).

Spatial correlation analysis of an additional set of nine randomly selected leaves showed similar relationships between bacterial α-diversity and leaf traits, despite variation in spatial distribution patterns among individual leaves (Supplementary Fig. 4–12). These results confirm the robustness of the patterns observed in Fig. 4. For the entire sampled leaves (*n* = 45), correlation analyses between bacterial α-diversity indices and leaf traits yielded consistent results (Table 3), further supporting the spatial findings and highlighting the value of hyperspectral proximal sensing for revealing leaf–bacteria interactions in the leaf microecosystem.

## Discussion

### Leaf trait models

Our results highlight the ability of leaf spectra to effectively capture specific leaf traits, while also indicating that this ability is strongly influenced by environmental conditions.

Abaxial spectra showed stronger correlations with structural traits such as leaf mass per area (LMA) and leaf water content (LWC), likely reflecting the anatomical asymmetry of oak leaves. The stomata-rich abaxial surface contrasts with the smoother, stomata-free adaxial side^47^, making structural and water-related variability more detectable in abaxial measurements. Meanwhile, stacking models yielded inconsistent results for structural traits, with more variable R^2^ values than PLSR, suggesting reduced model stability.

Sunlit versus shade spectra further revealed important differences, particularly for chemical traits such as chlorophyll (Chl) and nitrogen. Sunlit spectra consistently showed stronger correlations and R^2^ values comparable to previous studies^48,49^, whereas shade spectra showed greater.

variability, including negative R^2^ values in some cases, indicating that the model performed worse than simply using the mean of observed values as predictor. This difference may be attributed to the fact that sunlit leaves receive direct sunlight and are more actively photosynthesized^50^. Chl is a crucial parameter that directly affects photosynthetic efficiency and productivity^51^, while Nitrogen is linearly linked to the photosynthetic capacity because it is a key component of Rubisco, which constitutes a large proportion of the photosynthetic proteins^52,53^. Their active involvement in these physiological processes probably improves their detectability in the sunlit spectra.

Trait-specific challenges were also evident. Flavonoids (Flav) were difficult to predict across models, likely due to their strong absorption in the ultraviolet (UV) region, which falls outside the visible and near-infrared (NIR) ranges captured in our study. However, anthocyanins (Anth), a subclass of flavonoids, have the unique ability to absorb blue-green light in the visible region of the spec- trum^54,55^. This distinction is reflected in our results, where Anth exhibited stronger R^2^ values with the spectral data when compared to the overall Flav trait. As shown in (Supplementary Fig. 4a, b), the scatterplot and feature importance analysis for Anth prediction indicate that visible spectral region contributed significantly to the model’s performance.

### Leaf bacterial α-diversity models

Multiple bacterial indices were successfully predicted from leaf spectrum using our machine learning models. According to Fig. 3a, b, the feature importance peaks in the VIS region likely reflect biochemical influences — such as variation in carotenoids and xanthophylls — that shape bacterial richness^56^. In contrast, the 950–980 nm region suggests additional contributions from internal cell structural properties and hydration status^57^, which may significantly impact bacterial colonization. The correlations between bacterial richness and Chao1 indices were consistently stronger with shade leaf spectra than with those from sunlit leaves. This discrepancy likely reflects environmental constraints: sunlit leaves experience higher UV exposure and heat stress, which limit bacterial colonization and select stress-tolerant taxa, resulting in reduced diversity^58^. In contrast, shade leaves provide a more tempered microenvironment, supporting richer bacteria species, including a higher abundance of rare taxa^59^. Thus, the development of a more varied and dense bacterial layer in shade leaves^60^ may generate spectral signals that are captured more efficiently by hyperspectral imaging.

These ecological differences were reflected in our data (Fig. 3c, d). Shade leaves showed higher overall diversity and a more distinct gap between richness and Chao1 (152.5 vs. 172.3), suggesting a higher proportion of rare taxa. Sunlit leaves, in contrast, showed only a minor difference between the two indices (90.3 vs. 95.3), indicating a less species-rich community with fewer rare species. These trends also aligned with our model performance: while richness predictions were comparable across sunlit and shade spectra, Chao1 predictions were considerably more accurate with shade spectra, likely due to stronger spectral features associated with rare bacterial taxa. Rare taxa can signal both vulnerability and resilience under environmental change: they are vulnerable to heat/drought stress and some appear restricted to shaded microhabitats^61^. Yet when conditions shift from sunlit to shaded — and leaf physiological rates such as photosynthesis and transpiration decline — some rare taxa can become involved in specialized functions (e.g., nutrient-cycling steps), providing insurance effects for resilience^62–66^. The associated biochemical activity can generate distinct spectral signatures, consistent with the higher predictability of Chao1 from shaded spectra. By contrast, sunlit leaf spectra may be more strongly influenced by other physiological processes (e.g., pigment metabolism and water regulation), which could reduce correlation with bacterial diversity.

Furthermore, the consistently higher predictive performance of abaxial spectra compared to adaxial spectra for both richness and Chao1 reflects underlying structural and environmental asymmetries. Bacterial aggregations are frequently observed in intercellular grooves, trichomes, and stomata—structures that are often more abundant on the abaxial surface^67^. These topographical features may facilitate bacterial colonization, potentially enhancing the detectability of bacterial communities on the lower leaf surface. Additionally, environmental stressors, including UV radiation, temperature fluctuations, and desiccation, are generally more intense on the adaxial surface^68,69^.

Consistent with this view, independent ecological evidence shows fine-scale spatial heterogeneity of leaf-surface bacteria. Micrometer-scale (“fine-grained”) environmental heterogeneity is a key driver of microbial activity, diversity, distribution, and evolution^70,71^. Bioreporter studies show that bacterial colonization tracks the microscale distribution of resources and stresses on leaves —such as fructose, iron, water, UV, and phenolics — revealing pronounced patchiness at the single-cell scale^72–75^. In this context, our maps serve as model-based guidance to likely high-diversity zones, using hyperspectral indicators to prioritize regions aligned with known anatomical/resource micro- gradients (stomata, veins, trichome bases).

In most cases, the PLSR and stacking models performed similarly. This strong correlation highlights the potential of hyperspectral imaging to provide a deeper insight into the bacterial community diversity.

### Spatial predictions – unveiling the dependence of bacterial α-diversity on Spatial traits pattern on the leaf microecosystem

The spatial chlorophyll distribution maps showed a clear structure of the oak leaf. We compared the predicted Chl spatial map with the RGB image of the leaf. This comparison qualitatively indicated the consistency between spectral-based predictions and visible chlorophyll patterns. Both PLSR and stacking models effectively captured the general Chl distribution across the leaf, including detailed variations along the veins and edges.

Our models revealed an uneven spatial distribution of bacterial richness across the leaf surface, with consistently higher values toward the apex and reduced diversity at the base. Interestingly, this spatial clustering was consistent with previous microscale observations suggesting that bacterial aggregation on leaf surfaces is largely independent of taxonomic identity^8^. This pattern likely reflects underlying physiological gradients across the leaf that influence bacterial colonization. Nitrogen-rich regions, typically located near the leaf apex, may enhance bacterial establishment by supporting biosynthetic processes essential for growth, including the synthesis of proteins, nucleic acids, and other metabolites^53,76^. In contrast, anthocyanins and other secondary metabolites — often enriched at the leaf base and margins — are known to exhibit antimicrobial properties^77,78^, potentially inhibiting bacterial richness in these areas. These opposing effects are consistent with the Spearman correlation in Fig. 4e, where bacterial diversity positively tracked nitrogen availability and was inversely associated with anthocyanin content. Interestingly, chlorophyll content, although central to plant physiology, showed unclear association with bacterial richness-based metrics, indicating that photosynthetic function may not directly influence bacterial colonization.

It is worth highlighting that estimated rare bacterial taxa showed strong correlations with leaf N and Anth content (Fig. 4f, g). Although rare taxa represent only a small fraction of the bacterial community by abundance, they are capable of playing disproportionately significant roles^46^. Members of the rare biosphere are often functionally specialized, contributing to key biogeochemical processes such as nitrification, methylotrophy, or the degradation of trace compounds, many of which are tightly linked to nitrogen cycling^79^. It is suggested that these bacteria may act as regulators within bacterial networks, showing sensitivity to fine-scale chemical variation on the leaf surface^80^. As such, their association with nitrogen-rich zones could reflect microhabitats with elevated metabolic flux, while their exclusion from anthocyanin-rich areas may be driven by chemical defense barriers. These findings suggest that the spatial predictions of bacterial richness align with physiological gradients, thereby providing a biological basis for the reliability of our modeling approach.

Our findings highlight that interactions among phyllosphere bacteria, leaf traits, and leaf spectra are highly sensitive to changes in the ambient environment and leaf surface conditions. Furthermore, our novel findings on the correlations between leaf spectra and phyllosphere bacterial diversity provides insights into the capacity of leaf spectra in non-invasively detecting bacterial colonization. We provided further evidence that leaf spectra have the potential to characterize various leaf traits and detect plant physiological activities. Meanwhile, a comprehensive leaf-level analysis revealed the distribution of certain leaf traits and bacterial communities on the leaf surface is interdependent. These insights have direct implications for ecosystem-level GHG exchange under climate variability. Although trait-based remote sensing approaches are widely used to upscale plant physiological properties to canopy level^81–83^, bacterial dimensions remain largely unexplored in part because bacteria lack distinct spectral absorption features that are directly detectable in canopy reflectance spectra. Moreover, most microbe–GHG modeling frameworks have been developed for soils^84–86^. Our approach provides a path to extend such modeling to the phyllosphere, contingent on applying the technique across additional tree species and forest types, and subsequently upscaling to canopy and landscape scales. Accordingly, we propose that phyllosphere bacterial communities can be incorporated as a biological modifier within leaf-trait–based upscaling frameworks, thereby constraining model uncertainties and improving the representation of biotic controls on ecosystem GHG fluxes. To extend this work, we will also integrate ITS/18S metabarcoding with hyperspectral mapping to test whether the observed trait–bacteria relationships generalize to fungi and other eukaryotes, and to evaluate how these groups jointly shape leaf-scale processes. Moreover, while our pixel-level diversity maps are model-based inferences rather than direct within- leaf measurements, we provide a general framework for future spatially validation. Recent advances in fluorescence in situ hybridization (FISH) and adhesive tape-lift methods allow quantitative mapping of phyllosphere bacteria at the single-cell scale without introducing spatial bias^87^. Integrating such microscale imaging with multi-spot 16 S sequencing and qPCR within the same leaf — sampling predicted high- and low-diversity regions — would directly test our within-leaf predictions and calibrate the predicted spatial distribution of bacteria α-diversity.

**Table 1:** Prediction results for leaf traits using PLSR and stacking regression models in July (189 leaf samples) and September (188 leaf samples) across four predictor sets (sunlit, shaded, adaxial, abaxial).

July Season									
Leaf Trait	Model	Sunlit		Shade		Adaxial		Abaxial	
		R2	RMSE	R2	RMSE	R2	RMSE	R2	RMSE
Chl	PLSR Stacking	0.63 0.64	3.20 3.16	0.47 -0.31	5.07 8.01	0.48 0.00	4.26 5.89	-0.07 -0.13	4.61 4.74
Flav	PLSR Stacking	0.13 -1.01	0.33 0.50	0.19 -1.48	0.49 0.85	-0.23 0.23	0.54 0.43	0.48 0.23	0.33 0.40
NBI	PLSR Stacking	0.55 0.53	1.42 1.45	0.12 0.38	5.22 4.39	-0.18 0.07	3.31 2.94	0.22 0.12	2.32 2.47
Anth	PLSR Stacking	0.34 0.36	0.07 0.07	0.16 -0.22	0.07 0.09	0.24 0.16	0.07 0.08	0.33 0.13	0.06 0.06
FM	PLSR Stacking	0.34 -0.33	0.30 0.43	0.51 0.16	0.33 0.44	0.39 0.21	0.25 0.29	0.23 0.29	0.29 0.28
DM	PLSR Stacking	0.49 0.57	0.12 0.11	0.49 -0.49	0.15 0.25	0.06 -5.08	0.13 0.33	0.43 0.24	0.10 0.12
LMA	PLSR Stacking	0.27 0.13	0.00 0.00	0.43 -2.60	0.00 0.01	0.06 -3.01	0.00 0.01	**0.82** **0.78**	0.00 0.00
LWC	PLSR Stacking	0.42 0.43	2.23 2.22	0.22 -0.29	3.19 4.11	0.24 -2.48	3.71 7.94	**0.80** 0.65	1.96 2.58

September Season									
Leaf Trait	Model	Sunlit		Shade		Adaxial		Abaxial	
		R2	RMSE	R2	RMSE	R2	RMSE	R2	RMSE
Chl	PLSR Stacking	**0.74** **0.76**	3.33 3.25	0.15 0.19	4.31 4.21	0.43 0.47	4.47 4.28	0.45 0.44	4.34 4.42
Flav	PLSR Stacking	-0.01 -0.03	4.46 4.52	0.50 0.46	0.44 0.46	-0.39 -1.69	0.81 1.13	0.01 0.04	0.76 0.67
NBI	PLSR Stacking	0.50 0.49	1.42 1.43	0.38 0.19	3.22 3.68	0.41 0.39	2.21 2.24	0.30 0.19	2.47 2.65
Anth	PLSR Stacking	0.46 0.49	0.06 0.06	0.04 -0.19	0.08 0.09	0.08 -0.26	0.06 0.07	0.07 0.00	0.06 0.06
FM	PLSR Stacking	**0.74** 0.33	0.19 0.31	0.48 -1.84	0.41 0.97	0.57 -0.46	0.30 0.56	-0.06 -3.60	0.44 0.93
DM	PLSR Stacking	**0.72** 0.66	0.10 0.11	0.50 -3.30	0.12 0.51	0.69 0.69	0.11 0.11	0.14 -0.09	0.18 0.20
LMA	PLSR Stacking	0.32 0.20	0.00 0.00	0.68 0.65	0.00 0.00	0.62 0.60	0.00 0.00	**0.70** -1.80	0.00 0.01
LWC	PLSR Stacking	-0.38 -0.49	2.20 2.29	0.65 0.62	2.44 2.53	0.65 -1.74	2.29 6.74	0.65 0.69	2.53 2.16
N	PLSR Stacking	0.49 0.45	0.21 0.22	0.32 -1.40	0.31 0.56	0.05 0.05	0.33 0.33	0.26 0.33	0.30 0.29
C	PLSR Stacking	0.02 -0.28	2.76 3.27	0.14 0.32	0.84 0.75	-0.28 -1.44	0.90 1.21	-1.37 -2.23	1.11 1.27
C/N	PLSR Stacking	0.46 0.25	4.07 4.86	0.32 0.31	5.30 5.47	0.10 0.16	4.90 4.73	0.23 0.22	4.36 4.35

**Table 2:** Prediction results for bacterial variables using PLSR and stacking regression models (95 leaf samples) in all seasons across four predictor sets (sunlit, shaded, adaxial, abaxial).

Microbiome	Model	Sunlit		Shade		Adaxial		Abaxial	
		R2	RMSE	R2	RMSE	R2	RMSE	R2	RMSE
richness	PLSR Stacking	0.27 -0.06	31.87 38.36	**0.54** **0.51**	32.62 33.51	0.07 -0.08	44.56 48.20	0.17 0.31	45.90 41.75
Chao1	PLSR Stacking	0.40 0.12	33.94 41.16	**0.71** **0.56**	39.80 49.36	0.17 -0.02	63.16 70.26	0.25 0.19	62.78 65.21
Shannon	PLSR Stacking	-0.06 -0.01	0.53 0.52	-0.19 -0.30	0.59 0.62	-0.54 -0.65	0.59 0.61	-0.11 -0.29	0.49 0.53
invsimp	PLSR Stacking	0.30 0.33	9.21 8.97	-0.05 -0.05	11.23 11.23	-0.18 -0.36	11.52 12.39	-0.06 -0.07	10.23 10.29
Evenness	PLSR Stacking	0.40 0.39	0.08 0.08	-0.08 -0.31	0.11 0.12	-0.44 -0.72	0.12 0.13	0.38 0.35	0.09 0.09

**Table 3:** Average spearman correlation coefficients (ρ) between bacterial α-diversity indices and leaf traits, based on either predicted or observed values. For the predicted data, correlations were computed within individual leaves (pixel-wise) using machine learning–based predictions of both bacterial α-diversity indices (Richness and Chao1) and leaf traits, across ten randomly selected leaves; the values shown represent the average correlation across these leaves. For the observed data, correlations were calculated at the whole-leaf level using direct measurements of both bacterial α-diversity indices and leaf traits across 45 leaves.

Averaged correlation for pixel-wise analysis (10 leaves)		Correlation for all leaves (leaf based, *n* = 45)	
Predicted Trait	Richness Chao1	Observed Trait	Richness Chao1
Chl	0.03 0.13	Chl	-0.22 -0.25
NBI	0.28 0.40	NBI	0.44 0.34
Anth	-0.20 -0.35	Anth	-0.16 -0.11
N	0.41 0.52	N	0.41 0.35
C	-0.05 -0.12	C	-0.12 -0.19
C/N	-0.39 -0.53	C/N	-0.34 -0.29

## Supplementary Information

Below is the link to the electronic supplementary material.

Supplementary Material 1

## Data Availability

The dataset used during the current study is available from the corresponding author upon reasonable request.
