# Supplementary material for: Hyperspectral proximal sensing shows clear relation between Spatial pattern of leaf traits and bacterial alpha diversity
Source: Sci Rep. 2025 Dec 30;15:45803. doi: 10.1038/s41598-025-33183-4 (PMC12756227; doi:10.1038/s41598-025-33183-4)
Supplement: Supplementary file 1 — Supplementary Material 1 [file 41598_2025_33183_MOESM1_ESM.docx]

**Supplementary Materials**

**Supplementary Text 1: Interpretation of study area**

The study area is the Marburg Open Forest close to Caldern. The forest is a typical mixed beech-oak forest with various other tree spots (spruce, Douglas fir, etc.). It is located in the Lahn–Dill lower mountain ranges of Hesse (average altitude 263 m above sea level (ASL)), with an average slope angle of 32.5℃. The dominant predominant soil type is a Cambisol, the soil texture is silt loam. The soils have a lower available water capacity (80 mm) compared to other areas. The average annual air temperature (2 m above ground) of the forest for the period 2017-2024 is 9.4℃, with a mean relative air humidity of 86.4% and a mean solar irradiation of 119 W/m². The average annual rainfall of the area (weather station Cölbe close by) is 662.5 mm. The forest climate is varying with season and daytime, with average afternoon temperatures in summer reaching 25℃, an average irradiance of about 700 W/m² and a relative humidity below 60%. During the observation period, the lowest air temperatures below 0℃ were recorded in February (6-7 hours). Rainfall is equally distributed over the entire months, with minima in April and October for the observation period.

**
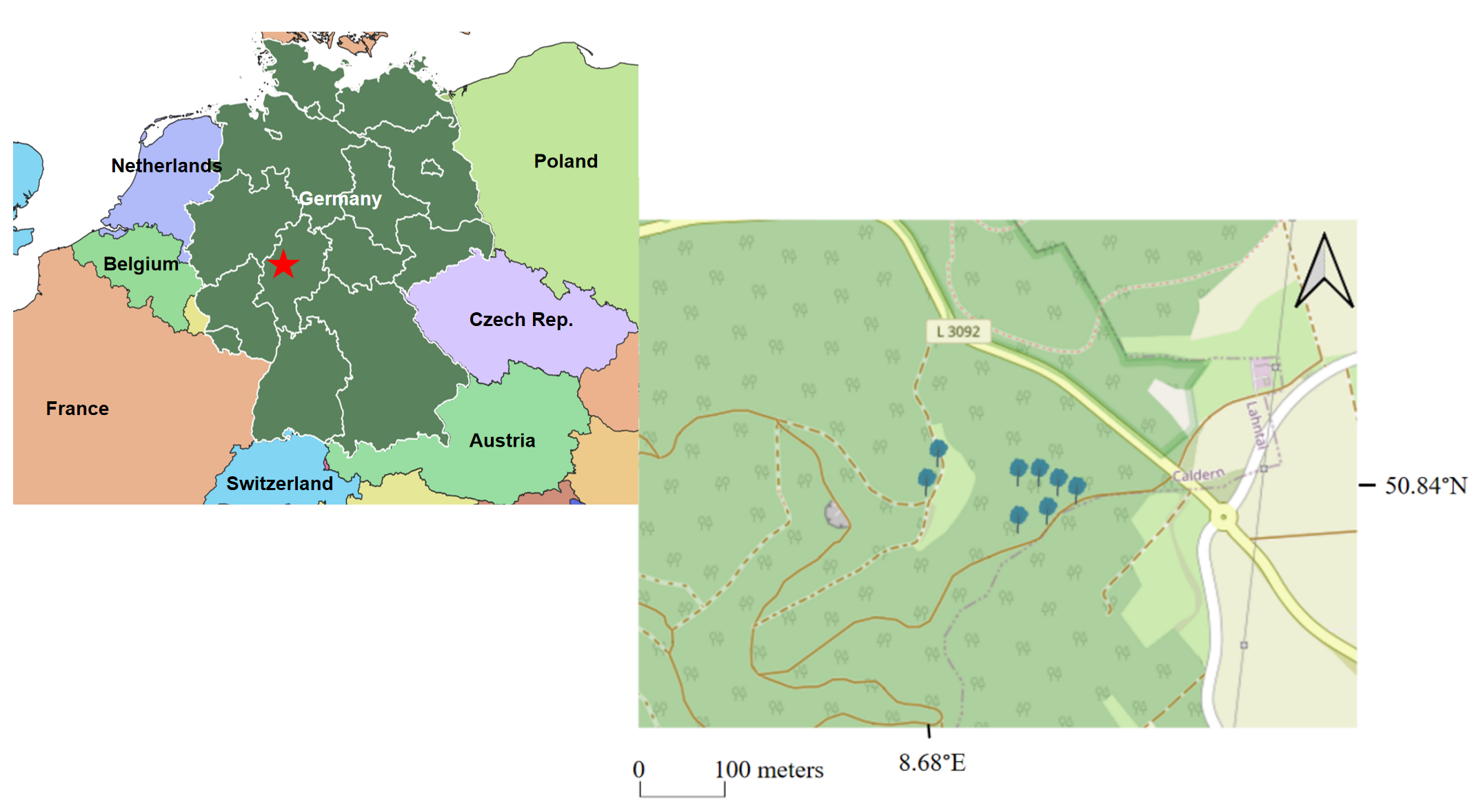
**

**Supplementary Fig. 1** Map of the location of the study area and the spatial distribution of the 8 selected Quercus robur trees used for leaf sampling. Administrative boundaries is provided by simplemaps.com. Basemap adapted from OpenStreetMap data, ©OpenStreetMap contributors, ODbL1.0 ([https://www.openstreetmap.org/copyright](https://www.openstreetmap.org/copyright?utm_source=chatgpt.com)). Produced work from OSM data; no OSM database is redistributed. Map composed by the authors in QGIS v3.36.0 ([https://qgis.org](https://qgis.org)).

**
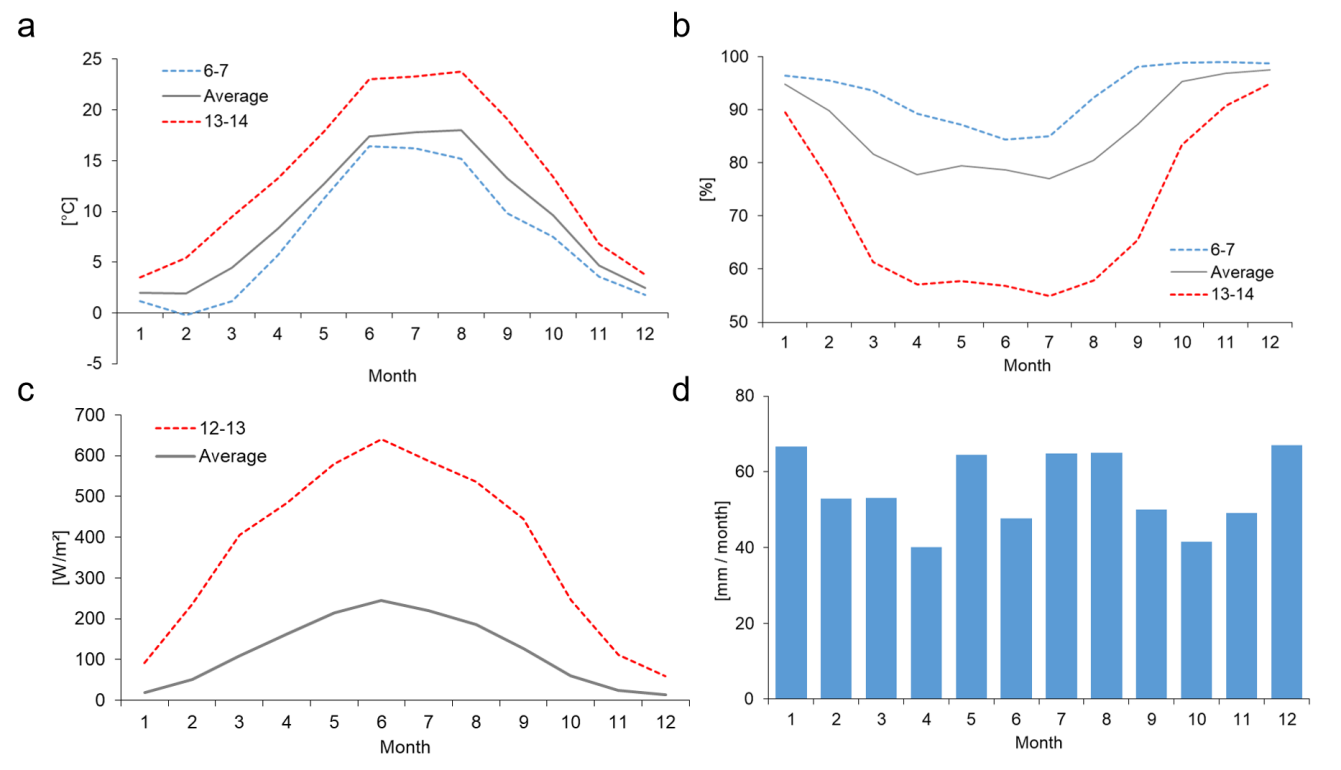
**

**Supplementary Fig. 2** Average climate of the study area from 2017 to 2024. **a,** Daily average of monthly air temperature at 2 m, including averages for 6–7 and 13–14 hours. **b,** Daily average of monthly air humidity at 2 m, with averages for 6–7 and 13–14 hours. **c,** Daily average of monthly global radiation at 2 m, with an average for 12–13 hours. **d,** Average monthly rainfall totals (662.5 mm per year). Air temperature, air humidity, and global radiation are observed at the automatic weather station “Grubenwiese” in the Marburg Open Forest (Longitude: 8.68477°E, Latitude: 50.8411°N, Altitude: 263.0 m ASL), while precipitation data is obtained from the Cölbe weather station nearby, maintained by the German Weather Service (Longitude: 8.7745°E, Latitude: 50.8492°N, Altitude: 187.0 m ASL).

| **Leaf traits** | **Unit** | **Microbiome indices** | **Unit** |
| --- | --- | --- | --- |
| Chl | ㎍/㎠ | Richness | Number of species |
| Flv | ㎍/㎠ | Chao1 | number of species |
| NBI | [Chl/Flav] | Shannon diversity index | - |
| Anth | ㎍/㎠ | inverse Simpson index | - |
| Leaf Area | ㎠ | Evenness | - |
| FM | g |  |  |
| DM | g |  |  |
| SLA | ㎠/g |  |  |
| LMA | g/㎠ |  |  |
| LWC | % |  |  |
| C | % |  |  |
| N | % |  |  |
| C/N | - |  |  |

**Supplementary Table 1** Leaf traits and microbiome diversity indices measured in this study as target variables for machine learning models, along with their respective units. Abbreviations: Chl = Chlorophyll content, Flv = Flavonoids, NBI = Nitrogen Balance Index, Anth = Anthocyanins, FM = Fresh Mass, DM = Dry Mass, SLA = Specific Leaf Area, LMA = Leaf Mass per Area, LWC = Leaf Water Content, C = Carbon content, N = Nitrogen content, C/N = Carbon-to-Nitrogen Ratio. Richness represents the total number of distinct microbial species within a leaf, while Chao1 accounts for rare species by estimating undetected species. The Shannon diversity index incorporates both species richness and evenness, using a weighted geometric mean of species proportions to measure diversity. The Inverse Simpson index estimates species dominance, where higher values indicate lower dominance and a more even species distribution. Evenness describes how uniformly individuals are distributed among species, with values closer to 1 indicating a more balanced distribution.

| **Correlation Strength** | **Nagative** | **Positive** |
| --- | --- | --- |
| None | -0.09 to 0.0 | 0.0 to 0.09 |
| Weak | -0.3 to -0.1 | 0.1 to 0.3 |
| Moderate | -0.5 to -0.3 | 0.3 to 0.5 |
| Strong | -1.0 to -0.5 | 0.5 to 1.0 |

**Supplementary Table 2**  Interpretation of correlation coefficients based on guidelines from Cohen et al.^36^ and Buda \& Jarynowski^37^.

**
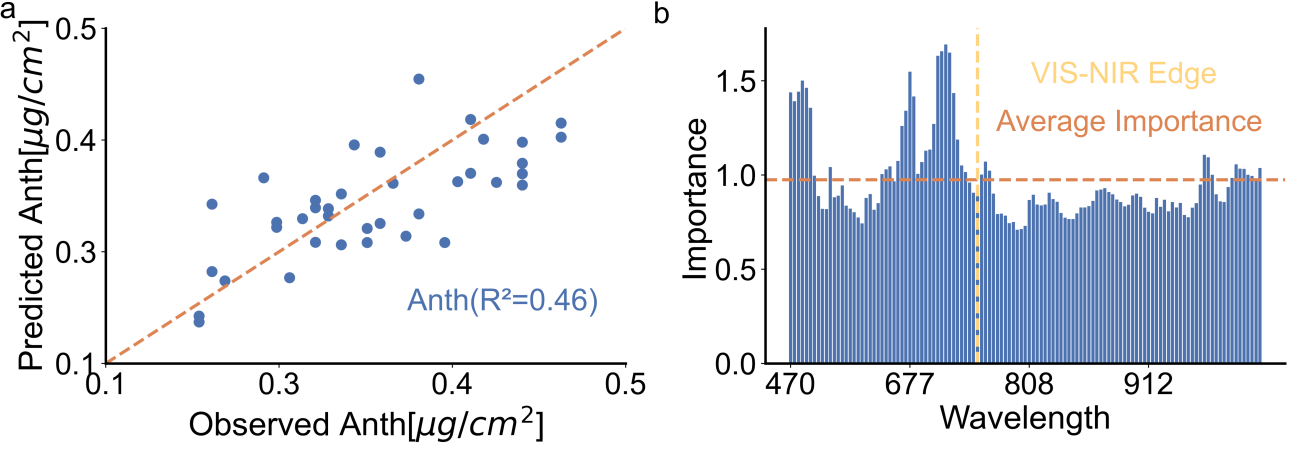
**

**
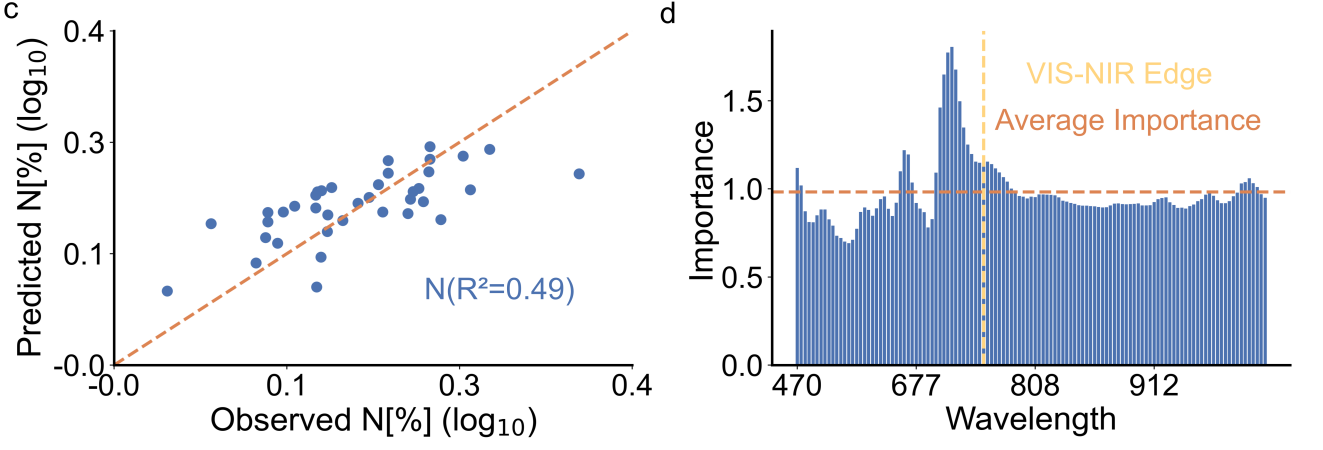
**

**Supplementary Fig. 3 a,b,** PLSR model predictions of anthocyanins(Anth, R^2^ = 0.46) using sunlit spectra in September. **c,d,** Nitrogen content (N, R^2^ = 0.49) using sunlit spectra in September. **a,c,** Scatterplot shows the relationship between observed and predicted values, with the orange dashed line representing the 1:1 relationship for reference. **b,d,** The feature importance plot indicates the contribution of different spectral regions to the prediction model. The blue bars represent the importance of individual spectral wavelengths to the model, with the orange dashed line indicating the average importance. The vertical yellow dashed line marks the boundary between the visible (VIS) and near-infrared (NIR) spectral regions.


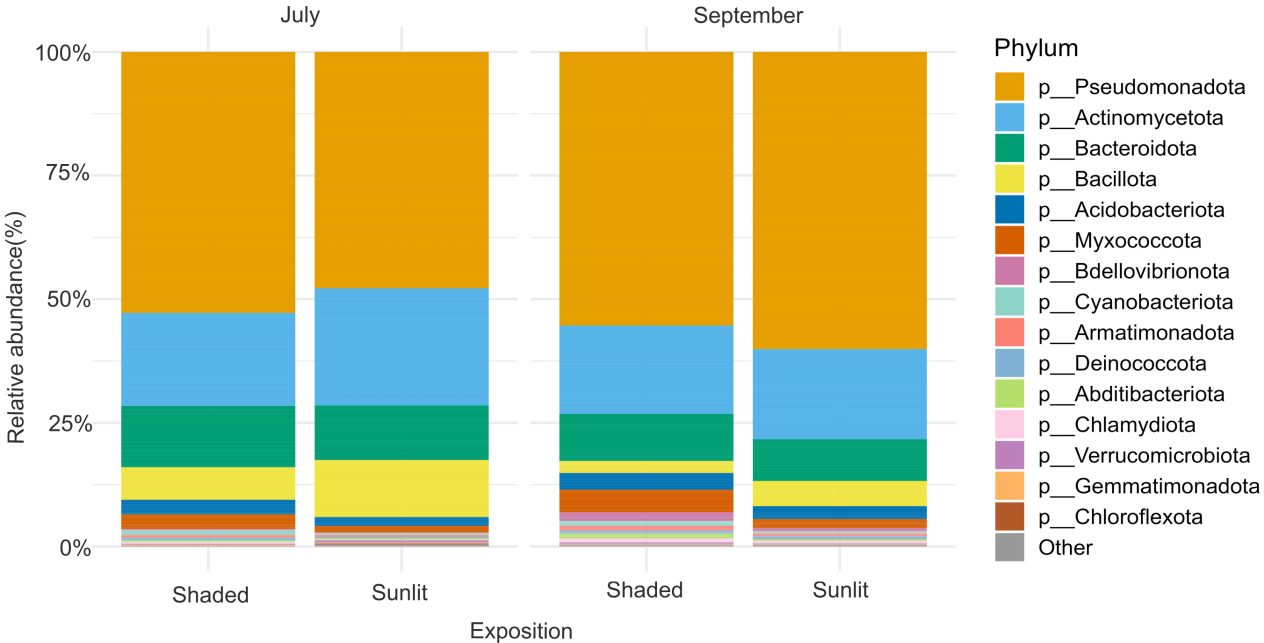


**Supplementary Fig. 4 :** Phylum level community structure of epiphytic leaf bacteria associated to Quercus robur, grouped by sampling month and leaf exposition. Bars are showing the 15 most abundant phyla, less abundant phyla are summarized as “Other”.

**
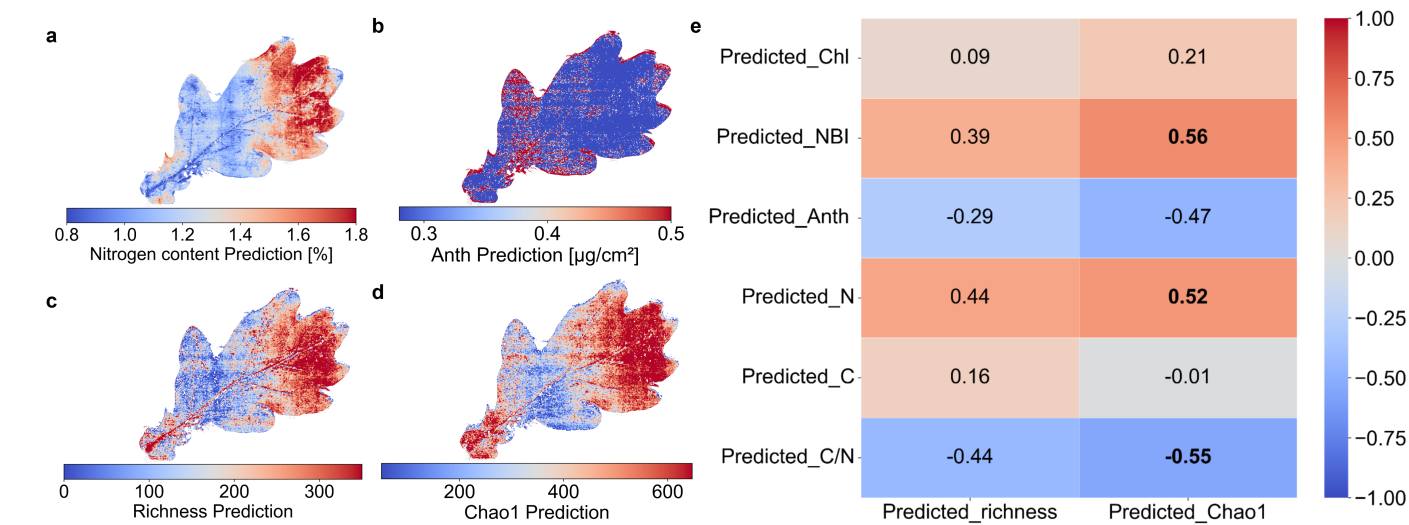
**

**Supplementary Fig. 5 :** Spatial correlation of leaf traits and microbial diversity in nine additional leaf samples. **a-d ,** Spatial maps of predicted nitrogen content, anthocyanin content, microbial richness, and Chao1 diversity index. **e,** Spearman correlation coefficients between predicted leaf traits (chlorophyll, nitrogen, carbon, C/N ratio, anthocyanins, and NBI) and microbial diversity indices (richness and Chao1). Maps generated in Python 3.11.9 (Jupyter; [https://jupyter.org/](https://jupyter.org/); Python: [https://www.python.org/](https://www.python.org/)). Key packages: scikit-learn 1.3.2, numpy 1.26.4, matplotlib 3.10.6, spectral (Spectral Python, SPy) 0.23.1.

All samples were collected in 2024 from Quercus robur (English oak) individuals at the Marburg Open Forest under natural field conditions. Notably, these samples were taken from the same trees used to train the predictive models based on 2023 data, ensuring consistency across sampling years. Sampling dates and tree identifiers corresponding to each figure are as follows:

· **Fig. 5**  – Tree 3, August 15th

· **Fig. 6**  – Tree 5, August 13rd

· **Fig. 7**  – Tree 2, September 16th

· **Fig. 8** – Tree 7, August 13rd

· **Fig. 9** – Tree 8, August 16th

· **Fig. 10** – Tree 8, August 16th

· **Fig. 11** – Tree 4, August 12nd

· **Fig. 12** – Tree 4, August 12nd

· **Fig. 13** – Tree 7, September 18th

**
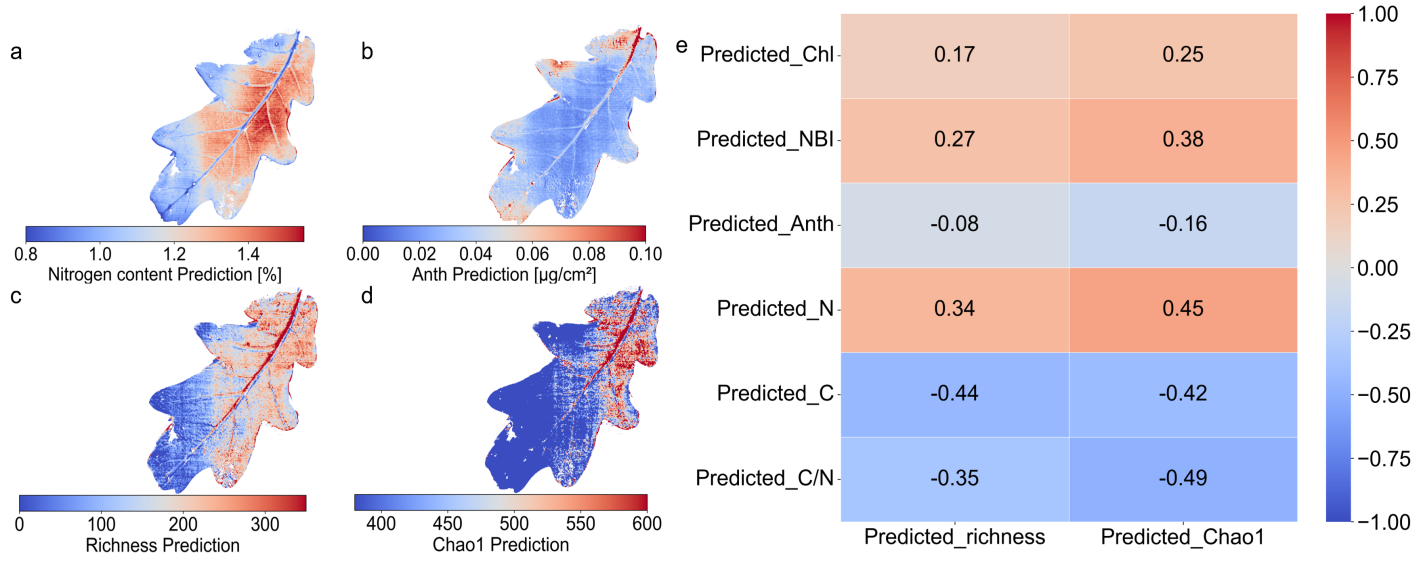
**

**Supplementary Fig. 6 :** Additional example from Tree 5 (August 13rd). Software and methods as in Supplementary Fig. 5.

**
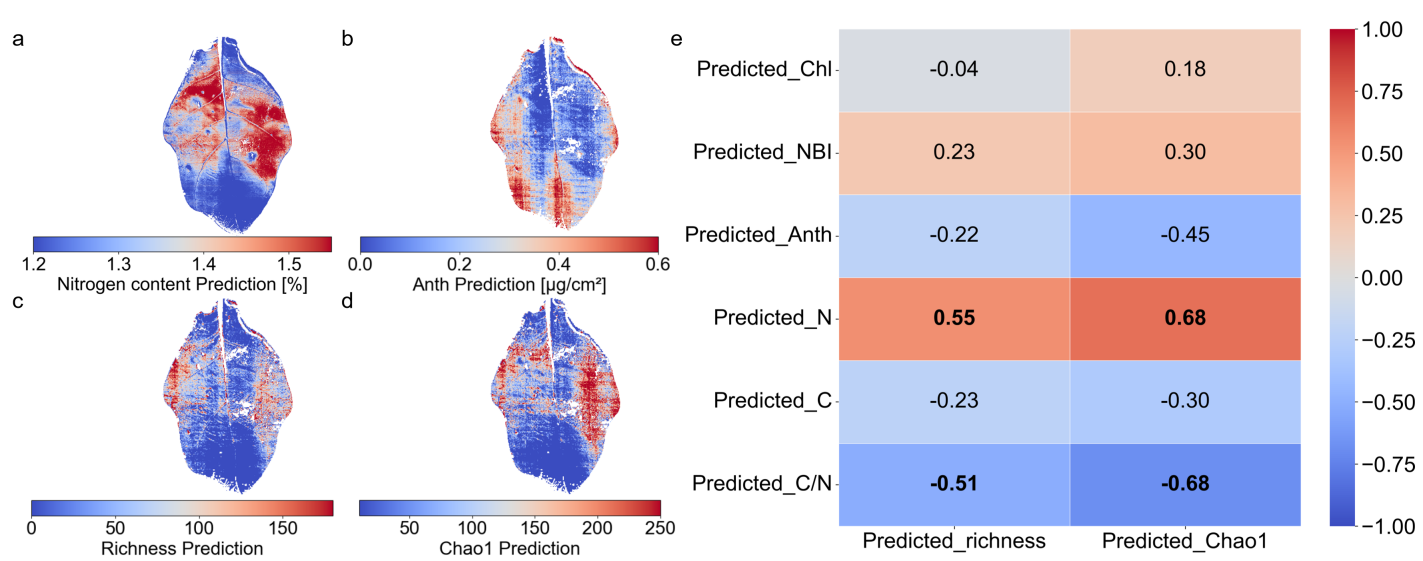
**

**Supplementary Fig. 7 :** Additional example from Tree 2 (September 16th). Software and methods as in Supplementary Fig. 5.

**
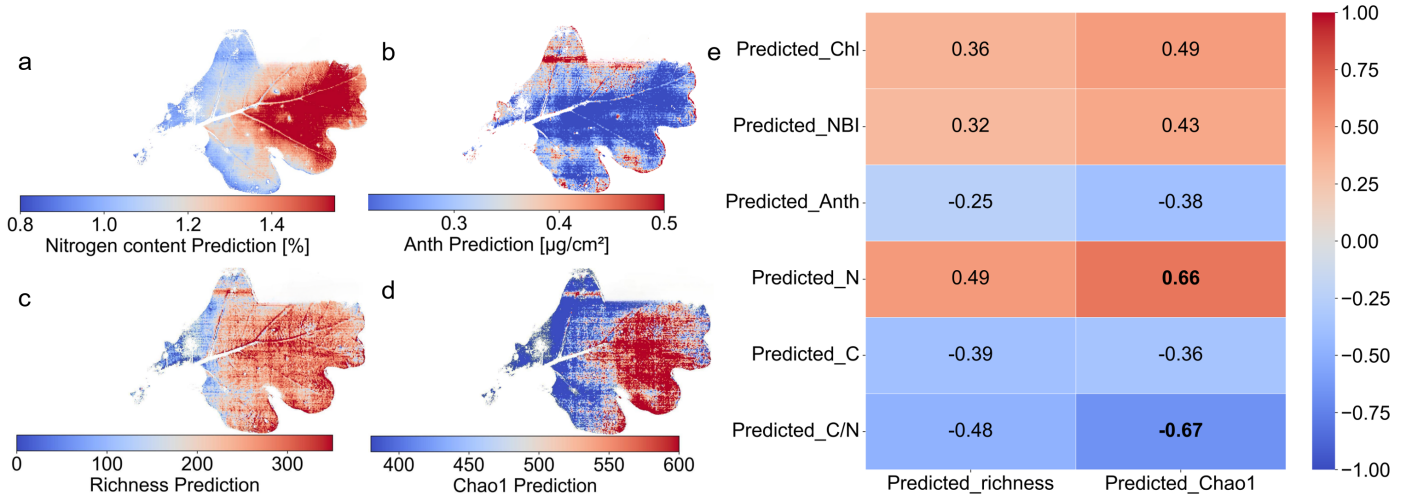
**

**Supplementary Fig. 8 :** Additional example from Tree 7 (August 13rd). Software and methods as in Supplementary Fig. 5.

**
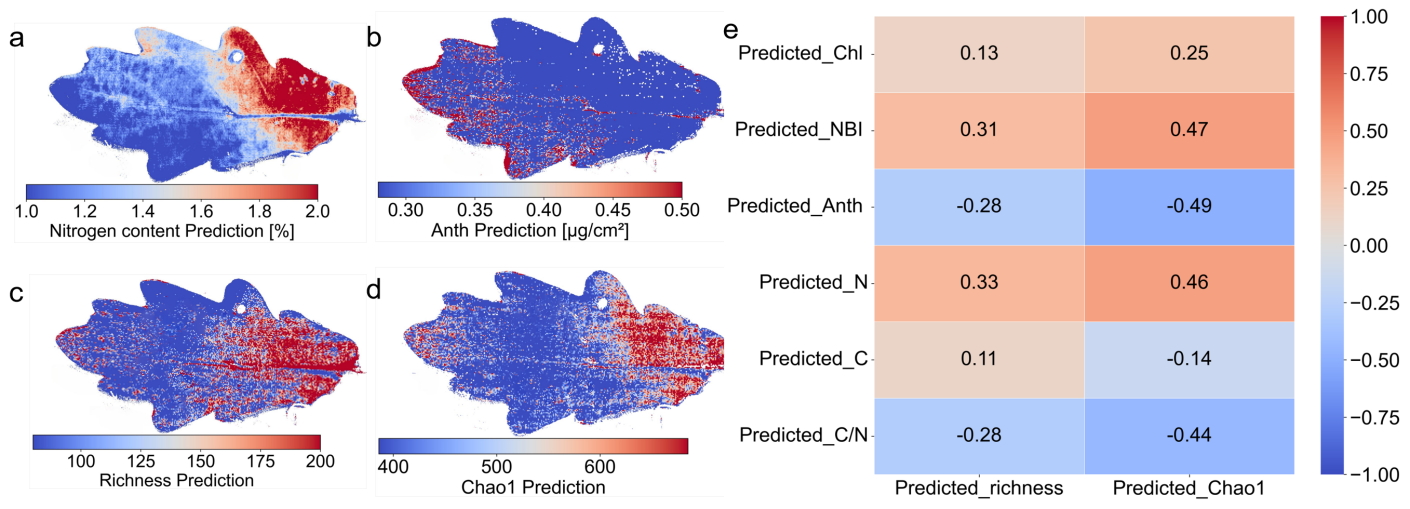
**

**Supplementary Fig. 9 :** Additional example from Tree 8 (August 16th). Software and methods as in Supplementary Fig. 5.

**
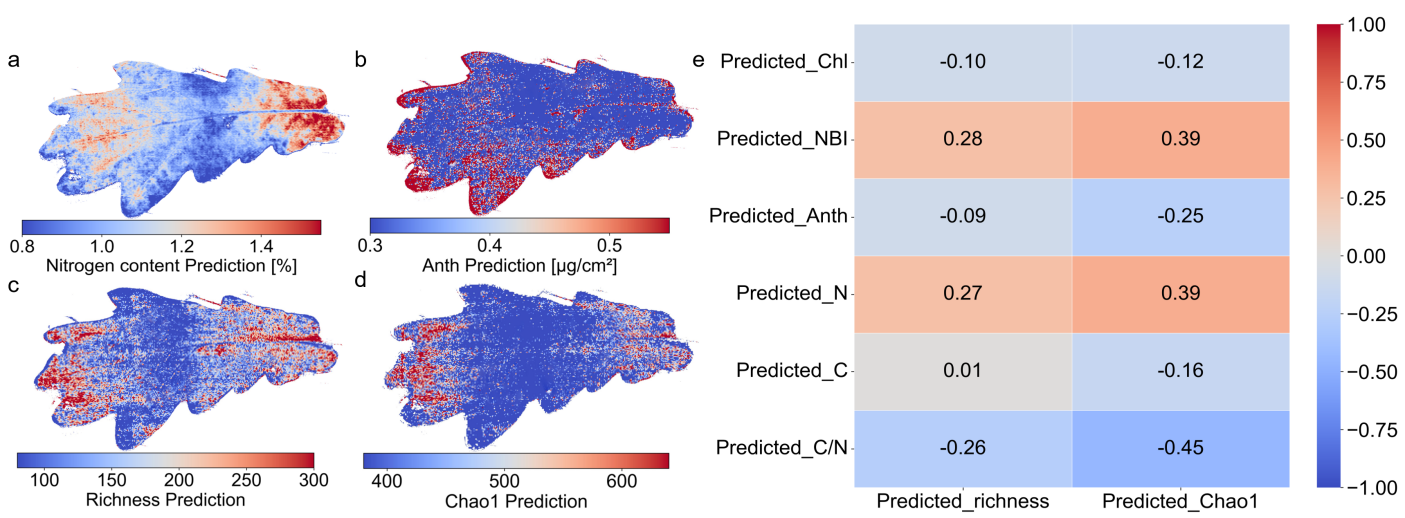
**

**Supplementary Fig. 10 :** Additional example from Tree 8 (August 16th). Software and methods as in Supplementary Fig. 5.

**
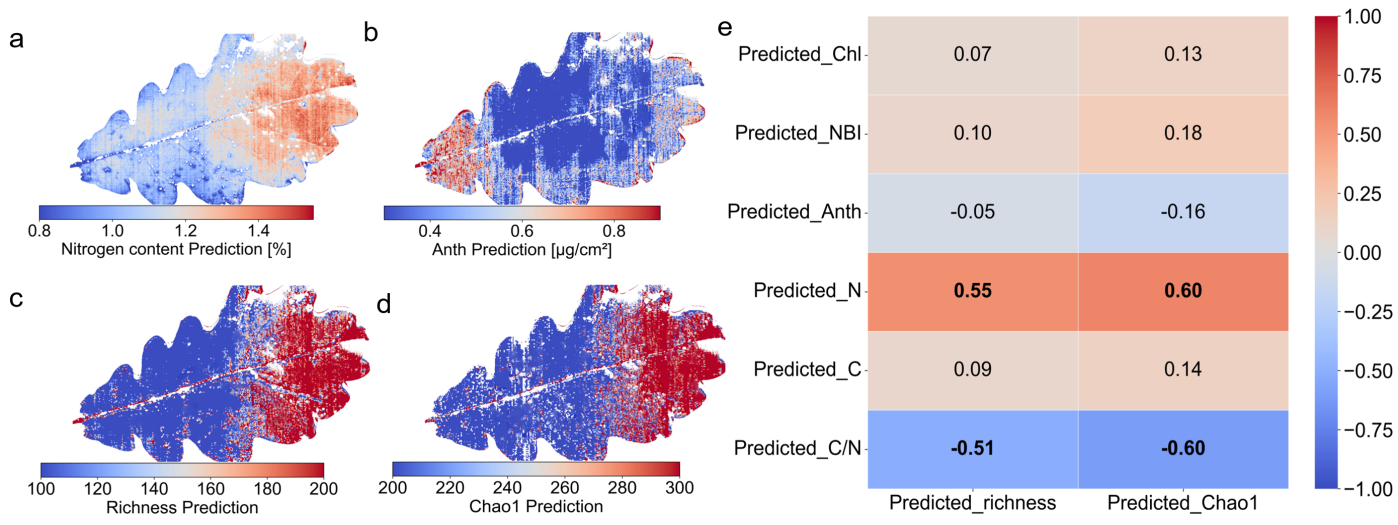
**

**Supplementary Fig. 11 :** Additional example from Tree 4 (August 12nd). Software and methods as in Supplementary Fig. 5.

**
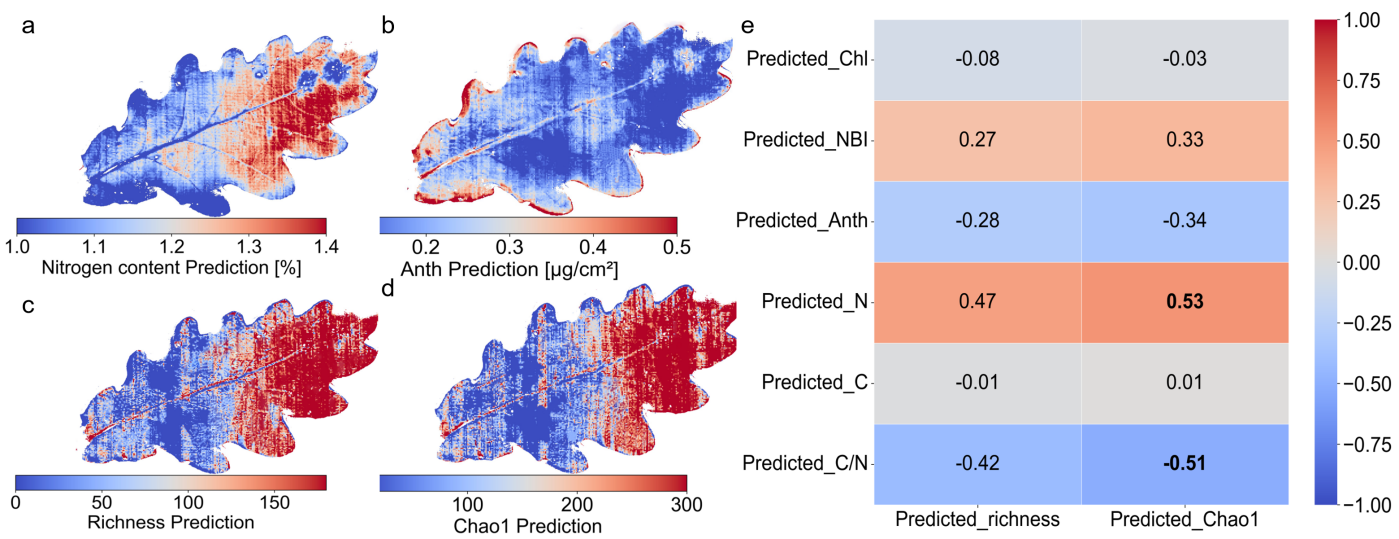
**

**Supplementary Fig. 12 :** Additional example from Tree 4 (August 12nd). Software and methods as in Supplementary Fig. 5.


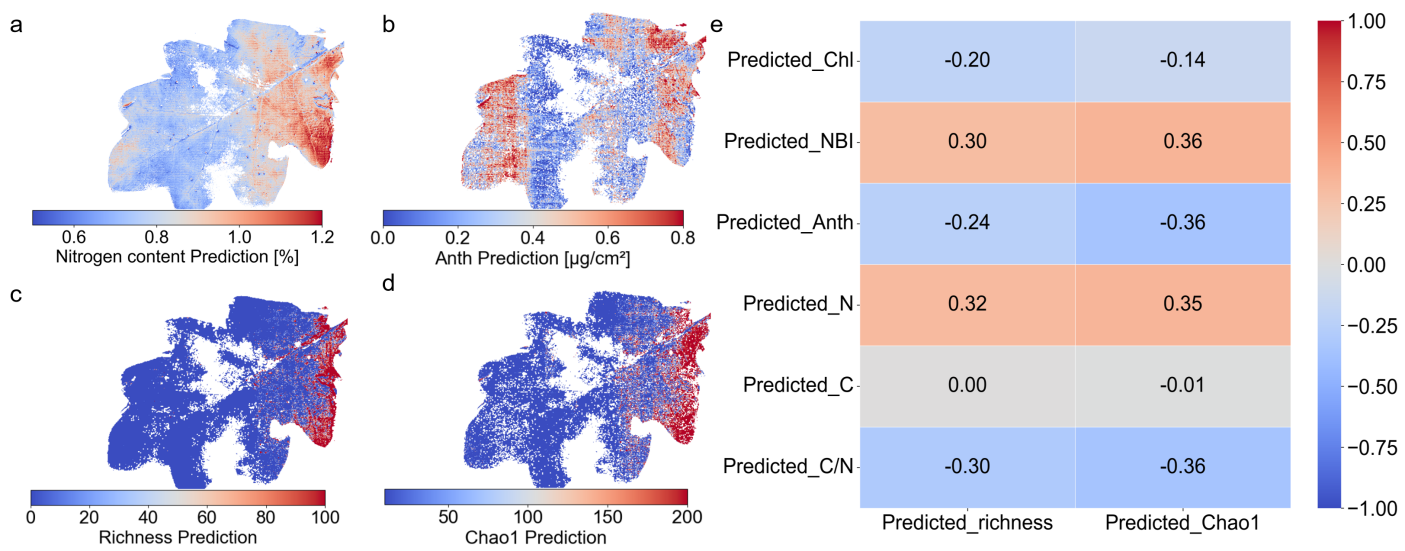


**Supplementary Fig. 13 :** Additional example from Tree 7 (September 18th). Software and methods as in Supplementary Fig. 5.
